# Usp18 deficient mammary epithelial cells create an antitumour environment driven by hypersensitivity to IFN-λ and elevated secretion of Cxcl10

**DOI:** 10.1002/emmm.201201864

**Published:** 2013-05-16

**Authors:** Christoph Burkart, Kei-ichiro Arimoto, Tingdong Tang, Xiuli Cong, Nengming Xiao, Yun-Cai Liu, Sergei V Kotenko, Lesley G Ellies, Dong-Er Zhang

**Affiliations:** 1 Moores Cancer Center, University of California San DiegoLa Jolla, CA, USA; 2 Division of Cell Biology, La Jolla Institute for Allergy and ImmunologyLa Jolla, CA, USA; 3 Department of Biochemistry and Molecular Biology, New Jersey Medical School, University Hospital Cancer CenterNewark, NJ, USA; 4 Department of Pathology, University of California San DiegoLa Jolla, CA, USA; 5 Division of Biological Sciences, University of California San DiegoLa Jolla, CA, USA

**Keywords:** breast cancer, Cxcl10, interferon-λ, UBP43, Usp18

## Abstract

The theory of cancer immunoediting refers to mechanisms by which the immune system can suppress or promote tumour progression. A major challenge for the development of novel cancer immunotherapies is to find ways to exploit the immune system's antitumour activity while concomitantly reducing its protumour activity. Using the PyVmT model of mammary tumourigenesis, we show that lack of the Usp18 gene significantly inhibits tumour growth by creating a tumour-suppressive microenvironment. Generation of this antitumour environment is driven by elevated secretion of the potent T-cell chemoattractant Cxcl10 by Usp18 deficient mammary epithelial cells (MECs), which leads to recruitment of Th1 subtype CD4^+^ T cells. Furthermore, we show that Cxcl10 upregulation in MECs is promoted by interferon-λ and that Usp18 is a novel inhibitor of interferon-λ signalling. Knockdown of the interferon-λ specific receptor subunit IL-28R1 in Usp18 deficient MECs dramatically enhances tumour growth. Taken together, our data suggest that targeting Usp18 may be a viable approach to boost antitumour immunity while suppressing the protumour activity of the immune system.

## INTRODUCTION

Novel effective treatments for cancer remain in high demand and targeting so called immunomodulators to enhance antitumour immunity has garnered considerable interest. Particular cells of the innate and adaptive immune system can, in conjunction with secreted molecules, function as potent extrinsic suppressors of tumourigenesis and cancer progression (reviewed in Vesely et al, 2011). However, depending on the type of tumour-infiltrating immune cells and established cytokine network the immune system can have an inhibitory or promoting effect on cancer growth. Extensive study of this dual role of immunity on tumourigenesis led to the development of the concept of cancer immunoediting. The hypothesis of immunoediting divides the interaction of the immune system with tumour cells into three phases: elimination (protection), equilibrium (persistence) and escape (progression) (Dunn et al, 2002, 2004; Shankaran et al, 2001). Each phase is associated with a typical immune cell population and cytokine network within the tumour. It is widely accepted that cytokines associated with a T helper cell subtype 1 (Th1) exhibit tumour-suppressive activity, whereas T helper cell subtype 2 (Th2) cytokines can promote tumour progression (Egeter et al, 2000; Haabeth et al, 2011; Muller-Hermelink et al, 2008; Shankaran et al, 2001). Therefore, an ideal immunotherapy approach will enhance the immune system's antitumour effect while maintaining or preferably decreasing its protumour effect.

One family of cytokines that has been associated with strong antitumour effects is the interferons (IFNs). A number of reports using transgenic animal models showed that mice deficient in IFNs or their receptors are more susceptible to spontaneous or drug-induced cancer (Dunn et al, 2005; Kaplan et al, 1998; Shankaran et al, 2001; Street et al, 2002) and many aggressive human cancers show impaired IFN signalling (Critchley-Thorne et al, 2009). However, IFNs have been widely discarded in current cancer therapy of solid tumours due to their broad effect on many different cell types resulting in severe side effects. Induction of the IFN-inducible gene Usp18 (also called Ubp43) leads to expression of two isoforms (Burkart et al, 2012). Both of these isoforms function as strong negative regulators of type I IFN signalling (Francois-Newton et al, 2011; Malakhova et al, 2006) and Isg15-specific de-conjugating enzymes (Malakhov et al, 2002). A role for Usp18 in the development of haematologic cancers was reported by two independent groups (Guo et al, 2010; Yan et al, 2007). Using different transgenic mouse models both reports show an inhibitory effect of Usp18 deficiency on leukaemia development. While the effect of Usp18 on solid tumour development has not been addressed, lack of Usp18 in the human breast cancer cell line MCF-7 leads to increased sensitivity to chemotherapy (Potu et al, 2010), suggesting that targeting specific IFN-inducible molecules may have therapeutic benefits. In this report, we chose a mouse model of spontaneous breast cancer to investigate the function of Usp18 in solid tumour development *in vivo*.

## RESULTS

### Reduction of mammary tumour growth in Usp18 null mice

**Figure 1:**
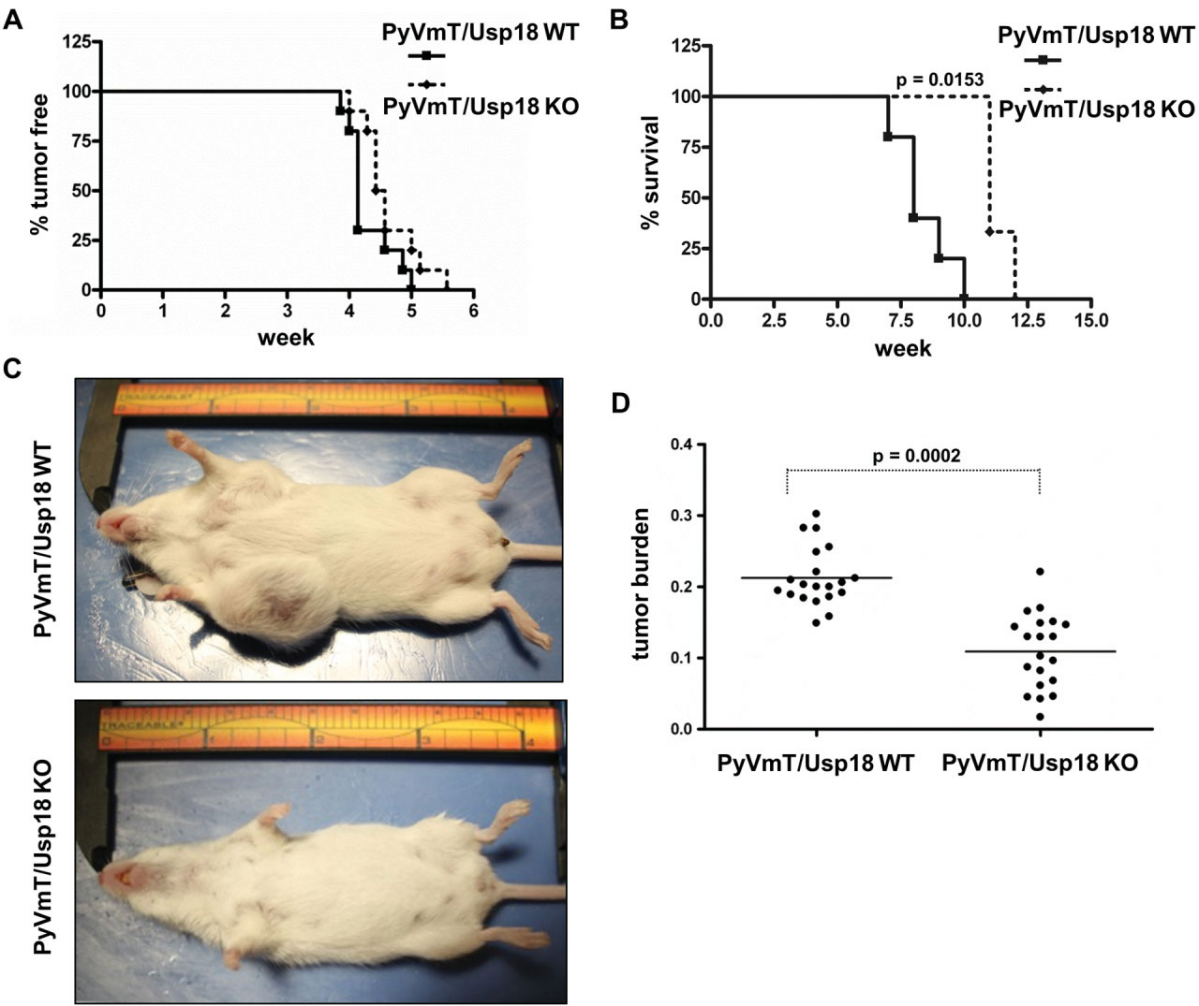
Usp18 deficient mice show decreased tumour burden in a murine breast cancer model Percentage of tumour-free mice shown by Kaplan–Meier plot. PyVmT/Usp18 WT and KO mice were palpated twice a week for mammary tumours. *n* = 10 for each cohort.Kaplan–Meier curves for survival of PyVmT/Usp18 WT and KO mice. A mean tumour diameter of 0.5 cm was used as endpoint for the survival studies. PyVmT/Usp18 WT, *n* = 5; PyVmT/Usp18 KO, *n* = 3.Representative photograph of a PyVmT/Usp18 WT and PyVmT/Usp18 KO mouse at 13 weeks of age.PyVmT mice were sacrificed at 13 weeks of age and tumour burden (tumour weight/body weight) determined. PyVmT/Usp18 WT, *n* = 20; PyVmT/Usp18 KO, *n* = 20.

To investigate the effect of Usp18 on mammary tumour development we used the polyomavirus middle T (PyVmT) mouse model for breast cancer (Guy et al, 1992). PyVmT/Usp18 knockout (KO) and PyVmT/Usp18 wild-type (WT) mice were generated by crossing the PyVmT and Usp18 deficient mouse lines. From week three onwards PyVmT transgenic female mice were palpated twice a week to monitor mammary tumour development. Tumour latency was not dramatically changed in PyVmT/Usp18 KO mice but a significant difference in survival between the two cohorts was observed (Fig 1). At 13 weeks of age when the PyVmT/Usp18 WT mice were terminated due to presence of multiple large tumours, PyVmT/Usp18 KO mice showed a clear reduction in size and number of visible tumours (Fig 1). Accordingly, the tumour burden was decreased by more than 50% in PyVmT/Usp18 KO mice (Fig 1).

### Lack of Usp18 inhibits angiogenesis and reduces invasiveness of mammary epithelial tumour cells

**Figure 2:**
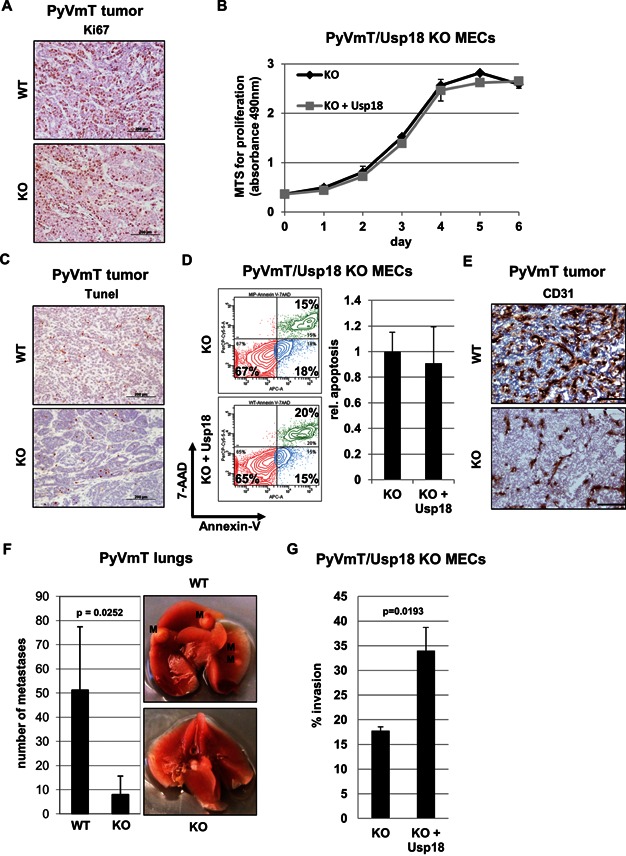
Deletion of Usp18 does not affect tumour cell proliferation or apoptosis but inhibits angiogenesis and invasiveness of tumour cells Characteristics of cancer cells were analyzed in PyVmT tumour tissues, and tumour cells isolated from PyVmT/Usp18 KO mice that were transduced with either pMSCV-puro (KO) or pMSCV-puro-HA-Usp18 (KO + Usp18). Paraffin-embedded tumour tissues were analyzed for proliferation marker Ki67 by immunohistochemistry. Images are 200× with 200 µm scale bar.Proliferative capacity of transduced primary tumour cells was analyzed *in vitro* by MTS assay.Number of apoptotic cells was determined with TUNEL assay on paraffin-embedded tumour tissues. Images are 200× with 200 µm scale bar.Percentage of apoptotic cells in transduced primary tumour cells was analyzed by AnnexinV staining (left panel) and relative apoptosis from three independent experiments determined (right panel).Immunohistochemical analysis of frozen tumour sections for angiogenesis marker CD31. Images are 200× with 200 µm scale bar.Number of spontaneous lung metastases in PyVmT mice of 13 weeks of age was determined by serial lung sections stained with H&E. *N* = 5 mice per group. Values shown represent mean total number of lung metastases ± SD (left panel). Representative photographs of lungs excised from PyVmT/Usp18 WT or PyVmT/Usp18 KO mice are shown (right panel). Macroscopically visible surface metastases are marked with “M.”Invasive potential of PyVmT/Usp18 KO MECs was determined in an *in vitro* assay using invasion chambers coated with matrigel. Shown are combined results of three independent experiments.If statistical significance was reached relevant *p* values are shown in the diagram.

To examine which characteristics of cancer cells are affected by Usp18 we analyzed tumours from PyVmT/Usp18 WT and PyVmT/Usp18 KO mice in more detail. In addition, for studying the role of Usp18 in mammary tumour epithelial cells, we also established mammary epithelial cell (MEC) lines derived from PyVmT/Usp18 KO tumours. These cell lines were transduced with either empty vector retrovirus (KO) or with Usp18 expression retrovirus (KO + Usp18). Levels of proliferation marker Ki67 were mostly unchanged in tumour tissues of PyVmT/Usp18 KO deficient mice (Fig 2). In concordance, the rate of cell proliferation was unchanged in an *in vitro* proliferation assay upon rescue of Usp18 deficiency (Fig 2) suggesting that lack of Usp18 does not have an intrinsic effect on proliferation of PyVmT MECs. Next, we addressed if the rate of apoptosis was altered in Usp18 deficient cells. Neither number of TUNEL-positive PyVmT/Usp18 KO tumour cells (Fig 2), nor the percentage of AnnexinV-positive stably transduced PyVmT/Usp18 KO MECs (Fig 2) was significantly different from controls, suggesting that the observed reduction in tumourigenesis is not due to elevated apoptosis. However, we did find a significant reduction in CD31 positive cells in PyVmT/Usp18 KO tumours, indicating an angiostatic effect of Usp18 deficiency (Fig 2). Interestingly, lack of Usp18 reduced the incidence of lung metastasis in PyVmT mice (Fig 2) that could be related to a decrease in invasiveness of cancer cells observed in *in vitro* matrigel invasion assays (Fig 2).

### Tumours of PyVmT/Usp18 deficient mice show increased CD4^+^ T-cell infiltration

**Figure 3:**
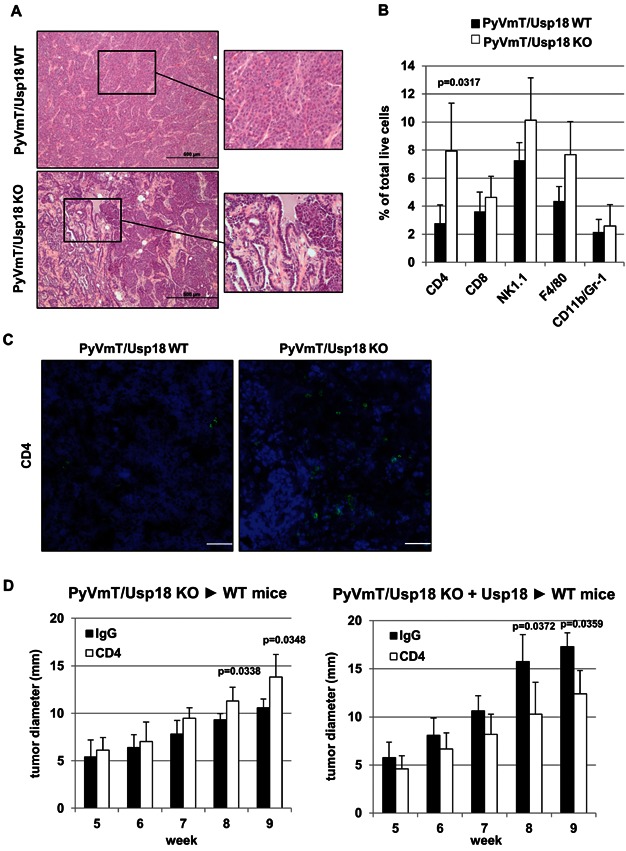
Histological and flow cytometric analysis show increased lymphocyte infiltration, particularly CD4^+^ T cells, into tumours of PyVmT/Usp18 KO mice H&E staining of paraffin-embedded mammary tumour tissue. Images are 100× with 500 µm scale bar.Single cell suspensions from tumours of PyVmT/Usp18 WT and PyVmT/Usp18 KO mice were analyzed for immune cell infiltration by flow cytometry. WT, *n* = 7; KO, *n* = 7.Frozen sections of mammary tumours stained for CD4^+^cells were analyzed by immunofluorescence. Images are 200× with 100 µm scale bar.Mice treated with either anti-CD4^+^antibody or control IgG were injected with PyVmT/Usp18 KO MECs or PyVmT/Usp18 KO + Usp18, respectively. Tumour growth was monitored weekly by measuring tumours with a calliper. Number of tumours analyzed: CD4, *n* = 6; IgG, *n* = 6.If statistical significance was reached relevant *p* values are shown in the diagram.

Analysis of Haematoxylin and Eosin (H&E) stained sections of mammary tumours from 13-week-old mice revealed a reduction in tumour progression in PyVmT/Usp18 KO mice. We distinguished early and late carcinoma from adenomas based on Lin et al's recommendations for the classification of mouse mammary tumour pathology (Lin et al, 2003). On average, mammary tumours of PyVmT/Usp18 KO mice showed a more adenoma-like pattern whereas PyVmT/Usp18 WT mice showed an early/late carcinoma pattern as demonstrated by loss of cellular architecture and sheet-like morphology (Fig 3). In order to identify and quantify the immune cells found in mammary tumours of PyVmT/Usp18 KO and PyVmT/Usp18 WT mice, we prepared single cell suspensions from tumours for flow cytometric analysis. We observed a significant increase in the number of CD4^+^ T cells in tumours of PyVmT/Usp18 KO mice compared to PyVmT/Usp18 WT mice (Fig 3). In addition, CD4^+^ T cells found in Usp18 KO tumours exhibited an enhanced activation status (Supporting Information Fig 1A). There was also a trend to an elevated number of CD8^+^ T cells, natural killer (NK1.1) cells and F4/80^+^macrophages in PyVmT/Usp18 KO tumours though the difference did not reach statistical significance. Tumour associated myeloid derived suppressor cells (CD11b^+^/Gr-1^+^), however, were not changed. We further confirmed an increase of CD4^+^ T cells in mammary tumours of Usp18 KO mice by immunofluorescence studies (Fig 3). Since we saw a bias towards CD4^+^ T cells in PyVmT/Usp18 KO tumours we investigated whether the total number of CD4^+^ T cells is elevated in Usp18 deficient mice. For this purpose, splenocytes from Usp18 KO and WT mice were isolated and analyzed for the number of CD4^+^ and CD8^+^ T cells. In contrast to the increased number of CD4^+^ T cells present in tumours of PyVmT/Usp18 deficient mice, we detected a small but significant decrease in splenic CD4^+^ T cells of Usp18 KO mice (Supporting Information Fig 1B). In order to test if CD4^+^ T cells play a protective role in an Usp18-dependent manner, we depleted FVB WT mice ofCD4^+^ T cells and then injected PyVmT/Usp18 KO MECs or PyVmT/Usp18 KO + Usp18 MECs into the mammary fat pad 2 days later. Mice received weekly injections of anti-CD4 antibody or control IgG and the efficiency of CD4^+^ T-cell depletion was confirmed by flow cytometric analysis (Supporting Information Fig 1C). CD4^+^ T-cell-depleted mice injected with PyVmT/Usp18 KO MECs showed significantly enhanced tumour growth compared to control IgG injected mice (Fig 3, left panel). Interestingly, removal of CD4^+^ T cells in mice injected with PyVmT/Usp18 KO + Usp18 MECs had a protective effect (Fig 3, right panel). This is in accordance with a report that used CD4^+^ T-cell depletion in a similar breast cancer transplantation model and demonstrated delayed tumour growth in the absence of CD4^+^ T cells (Yu et al, 2005). Based on these findings, we hypothesized that Usp18 regulates the pro- and antitumoural effect of CD4^+^ T cells and that the higher number of CD4^+^ T cells found in PyVmT/Usp18 KO tumours is due to elevated levels of one or more chemokines that attract T cells.

### PyVmT/Usp18 KO MECs secrete elevated levels of Cxcl10 creating a Th1/M1-polarized cytokine tumour environment

**Figure 4:**
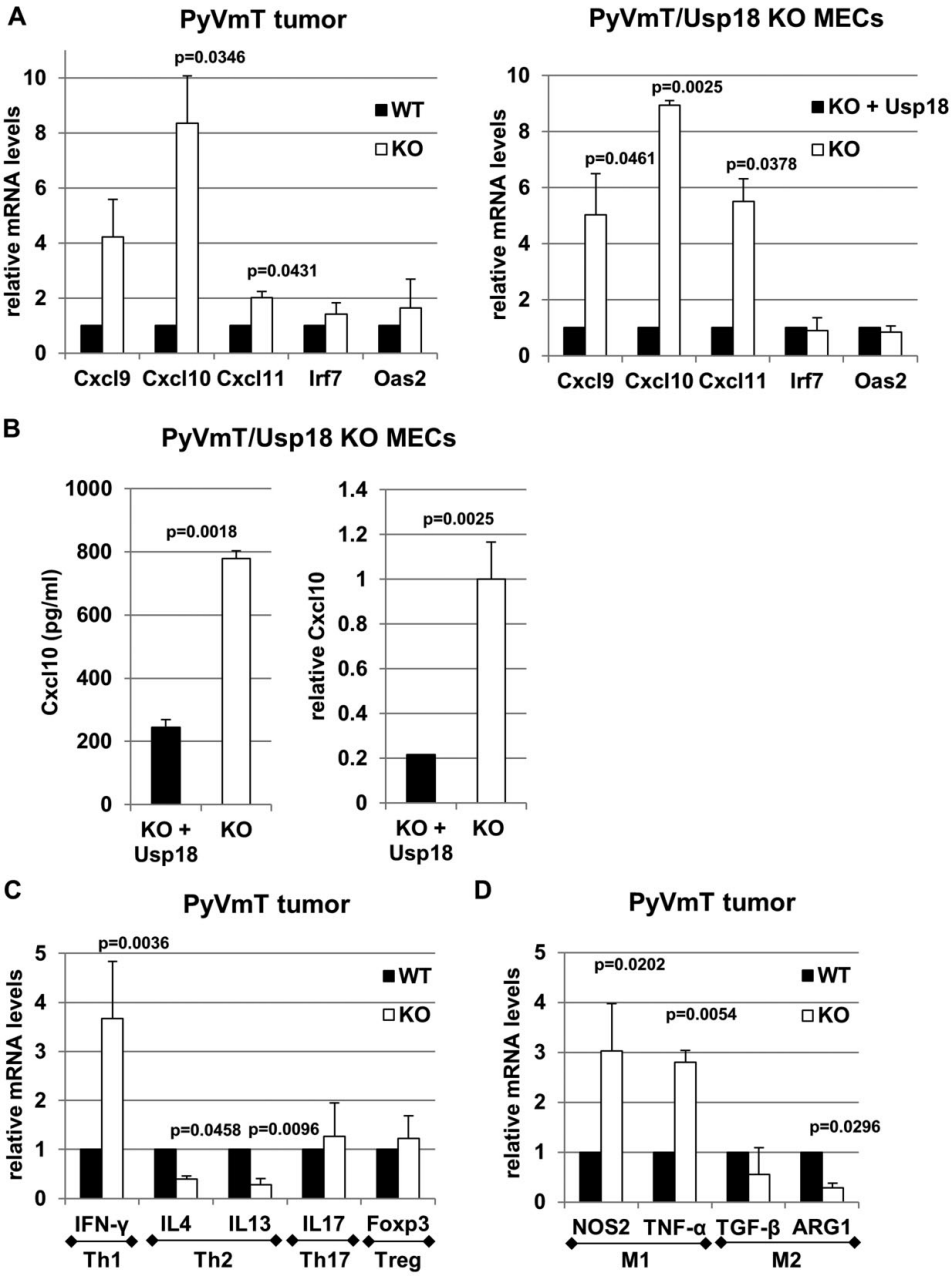
PyVmT/Usp18 KO mammary epithelial cells express and secrete elevated levels of the T-cell chemoattractant Cxcl10 creating a Th1-like cytokine microenvironment Transcript levels of Cxcr3 ligands and IFN genes Irf7 and Oas2 were analyzed by qRT-PCR in both tumours and transduced PyVmT/Usp18 KO MECs. The relative means from two separate tumours per genotype normalized to WT (left panel) or relative means from three independent experiments normalized to Usp18 (right panel) are shown.Cxcl10 protein levels in the culture medium supernatant of transduced PyVmT/Usp18 KO MECs were determined by ELISA. Left panel shows representative experiment with total values and right panel shows relative Cxcl10 levels from three independent experiments.Cytokines in the tumour microenvironment associated with T-cell subtypes were analyzed by qRT-PCR. Mean values of two separate tumours per genotype normalized to WT are shown. Experiment was performed in triplicate.Cytokines in the tumour microenvironment associated with macrophage subtypes were analyzed by qRT-PCR. Mean values of two separate tumours per genotype normalized to WT are shown. Experiment was performed in triplicate.If statistical significance was reached relevant *p* values are shown in the diagram.

Since the total number of CD4^+^ T cells is not elevated in Usp18 KO mice we examined whether T-cell-specific chemokines were upregulated in PyVmT/Usp18 KO tumours. The IFN-inducible Cxcr3 ligands Cxcl9, Cxcl10 and Cxcl11 are known potent chemoattractants for T cells and to a lesser degree for monocytes and natural killer (NK) cells (Khan et al, 2000; Thomas et al, 2003; Xie et al, 2003). Analysis of tumours from PyVmT mice and cultured PyVmT MECs showed that transcript levels of all three Cxcr3 ligands were elevated in the absence of Usp18 (Fig 4). Only Cxcl10 and Cxcl11, however, showed statistically significant upregulation in both PyVmT tumours and cultured MECs. Remarkably, no significant upregulation of other IFN-inducible genes such as Irf7 or Oas2 was observed in the PyVmT tumours or MECs tested. Since Cxcl10 was the most highly upregulated gene among the three and is considered to be the functionally dominant Cxcr3 ligand (Dufour et al, 2002; Hsieh et al, 2006), we focused on this chemokine in further studies. First, we confirmed that elevated transcription of Cxcl10 led to increased Cxcl10 levels in the medium of *in vitro* cultured Usp18 deficient PyVmT MECs by ELISA (Fig 4). Since expression of the chemokine receptor Cxcr3 is associated with CD4^+^ Th1 cells (Loetscher et al, 1996; Yamamoto et al, 2000), we examined whether tumours of PyVmT/Usp18 KO mice exhibit a Th1-like cytokine profile. Our studies revealed that the Th1 cytokine IFN-γ was increased and the Th2 cytokines IL4 and IL13 were decreased in PyVmT/Usp18 deficient tumours (Fig 4). Expression of genes known to be related to Th17- or Treg-subtype polarization such as IL17 or Foxp3 were unchanged between the two. In accordance with the observed Th1 bias, expression of genes characterizing M1 macrophage polarization was also elevated in tumours of PyVmT/Usp18 deficient mice (Fig 4).

### Usp18 deficient MECs are mediating the observed antitumour effect and elevated secretion of Cxcl10 is a driving force

**Figure 5:**
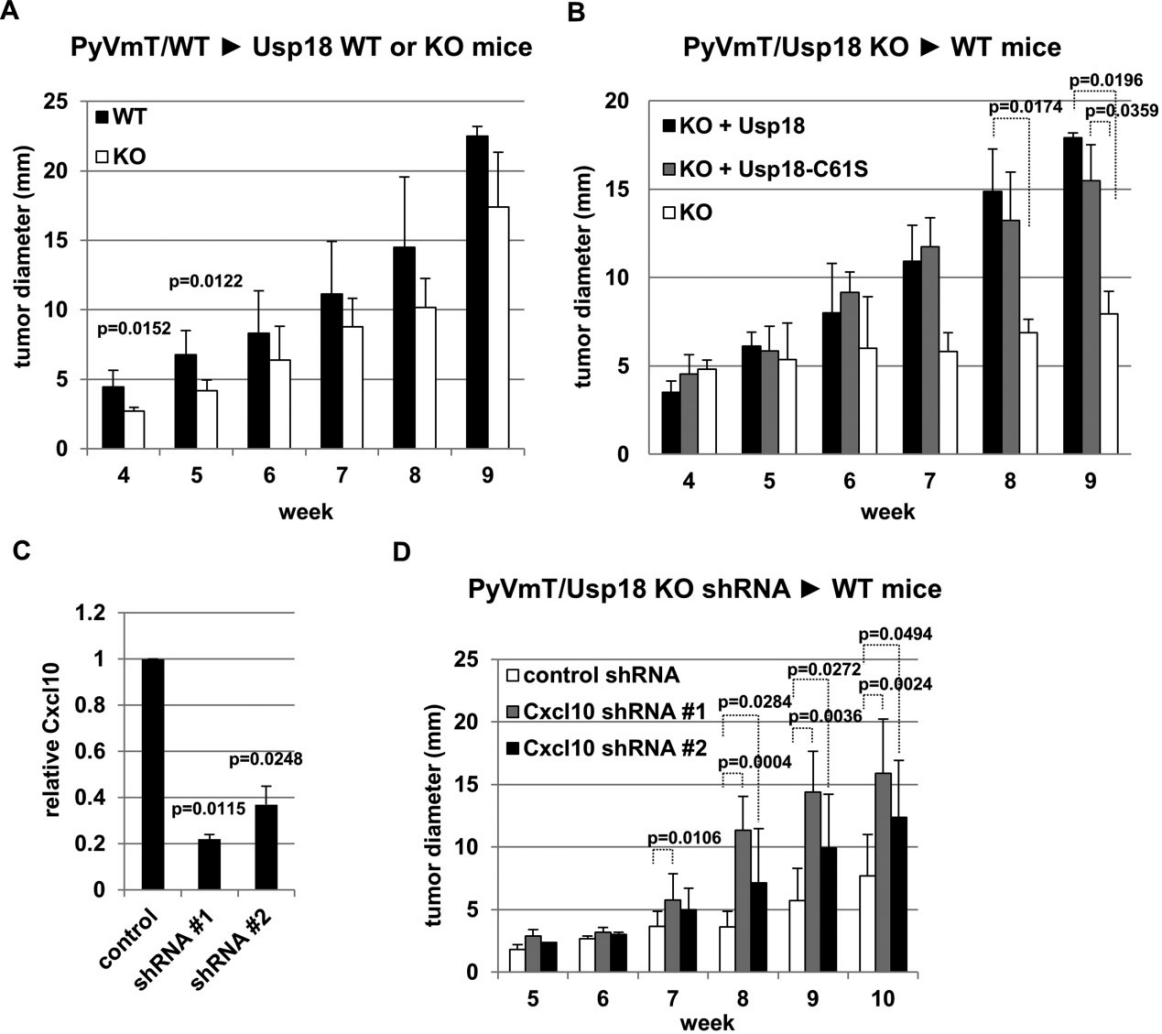
Antitumour effect of USP18 deficiency is due to extrinsic effects mediated by MECs and knockdown of Cxcl10 in PyVmT/Usp18 KO MECs promotes tumour progression Wild-type PyVmT/MECs were injected into two mammary fat pads per Usp18 KO or WT mouse. Tumour growth was monitored weekly by measuring tumours with a calliper. Number of tumours analyzed: WT, *n* = 8; KO, *n* = 7.Control vector (KO), Usp18 (KO + Usp18) or Usp18-C61S (KO + Usp18-C61S) transduced PyVmT/Usp18 KO MECs were injected into two mammary fat pads per WT mouse. Tumour growth was monitored weekly by measuring tumours with a calliper. Number of tumours analyzed: KO, *n* = 8; KO + Usp18, *n* = 8; KO + Usp18-C61S, *n* = 7.Cxcl10 protein levels in the culture medium supernatant of Cxcl10 knockdown PyVmT/Usp18 KO MECs was determined by ELISA. Experiment was performed in triplicate.PyVmT/Usp18 KO MECs transduced with control or Cxcl10 shRNAs were injected into two mammary fat pads per wild-type mouse. Tumour growth was monitored weekly by measuring tumours with a calliper. Number of tumours analyzed: Control shRNA *n* = 10, Cxcl10 shRNA#1 *n* = 10, Cxcl10 shRNA#2 *n* = 9.If statistical significance was reached relevant *p* values are shown in the diagram.

Our data suggested that Usp18 deficient PyVmT MECs altered the tumour microenvironment by elevated secretion of chemokines that promote a Th1-like cytokine network. However, at this point we could not exclude the possibility that Usp18 deficient stromal cells are responsible for the observed phenotype in our breast cancer model, since we used conventional systemic PyVmT/Usp18 knockout mice. To discriminate between tumour stroma and MEC effect we used a syngeneic tumour cell injection model. Injection of PyVmT/Usp18 WT MECs into the mammary fat pad of Usp18 KO and WT mice showed that the rate of tumour progression was not significantly changed between the two, suggesting no dramatic antitumour effect mediated by Usp18 deficient stromal or immune cells in the presence of PyVmT/Usp18 WT MECs (Fig 5). In contrast, injection of PyVmT/Usp18 KO MECs transduced with control vector led to significantly impaired tumour growth compared to Usp18 rescued PyVmT/Usp18 KO MECs or Usp18-C61S mutant rescued PyVmT/Usp18 KO MECs (Fig 5). Usp18-C61S is the catalytically inactive mutant form of Usp18 that lacks the ability to remove ISG15 from ISGylated proteins (Supporting Information Fig 2A). The fact that orthotopic injection of PyVmT/Usp18 KO + Usp18 and PyVmT/Usp18 KO + Usp18-C61S MECs led to similar tumour growth rates suggests that protein ISGylation or at least the difference of total protein ISGylation reached in this system does not contribute to establishment of an antitumour environment by Usp18 KO MECs. To strengthen the physiological relevance of our model we also confirmed that levels of reconstituted Usp18 in PyVmT/Usp18 KO MECs do not exceed maximum levels of endogenous protein reached upon induction (Supporting Information Fig 2B). We also compared tumour growth in WT mice injected with PyVmT/WT or PyVmT/Usp18 KO + Usp18 MECs and did not detect a significant difference between the two (Supporting Information Fig 2C), further showing that rescued Usp18 KO MECs behave similar to WT MECs in this model. Next, we aimed to determine the importance of elevated Cxcl10 expression for the reduced tumour growth inPyVmT/Usp18 deficient mice. To do so we stably transduced PyVmT/Usp18 KO MECs with control or Cxcl10 shRNA and injected these cell lines into the mammary fat pad of WT mice to determine tumour growth rates. In the generated cell lines, the efficiency of Cxcl10 knockdown was about 60–80% as determined by ELISA (Fig 5). Two separate cell lines expressing two different Cxcl10 specific shRNAs showed more aggressive tumour growth than the cell line expressing control shRNA, underlining the importance of elevated Cxcl10 expression in the observed phenotype of PyVmT/Usp18 KO mice (Fig 5). Additional analysis of tumours derived from MECs transduced with Cxcl10 shRNA confirmed the expected decrease in number of total leukocytes (CD45^+^) and CD4^+^ T cells present in the tumour (Supporting Information Fig 3A). Furthermore, immunohistochemical analysis only showed a slight increase in proliferation and number of apoptotic cells, but significantly increased neovascularization in tumours with reduced Cxcl10 levels (Supporting Information Fig 3B).

### Hypersensitivity of PyVmT/Usp18 KO MECs to IFN-λ enhances upregulation of Cxcl10 expression and inhibits tumour progression

**Figure 6:**
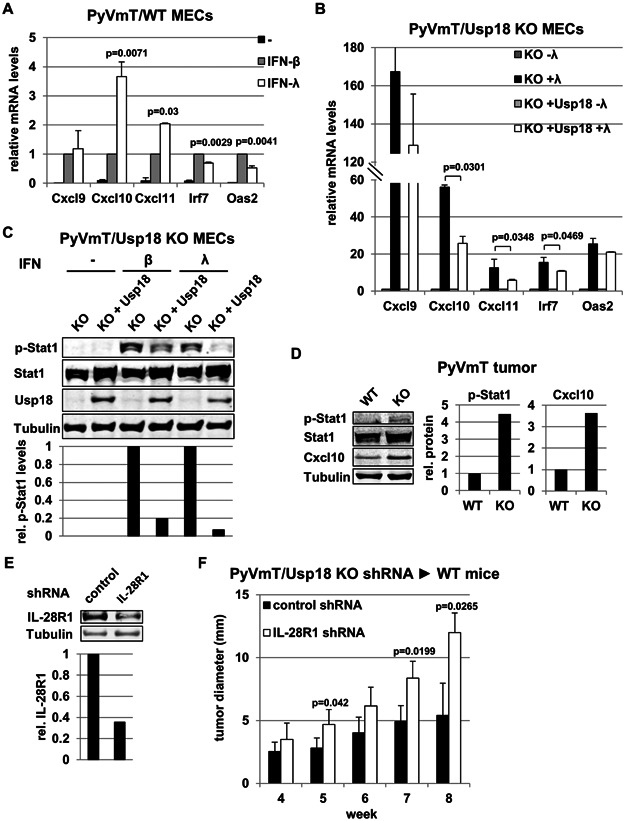
Hypersensitivity of PyVmT/Usp18 KO MECs to IFN-λ enhances Cxcl10 expression and protects against mammary tumour progression Source data is available for this figure in the Supporting Information. Induction of Cxcr3 ligands, Irf7 and Oas2 upon IFN-β or IFN-λ treatment in parental MECs was analyzed by qRT-PCR. Relative means from three independent experiments normalized to IFN-β treated cells are shown.Induction of Cxcr3 ligands, Irf7 and Oas2 upon IFN-β or IFN-λ treatment in transduced PyVmT/Usp18 KO MECs was analyzed by qRT-PCR. Relative means from three independent experiments normalized to untreated cells are shown.Phosphorylation of StatT1 in control vector (KO) or Usp18 (KO + Usp18) transduced PyVmT/Usp18 KO MECs upon IFN-β or IFN-λ treatment was determined by Western blotting. Cells were left untreated or IFN treated for 15 min before analysis. Cells were harvested, lysed and analyzed for Usp18, Stat1 and phosphorylated Stat1 levels by Western blotting. Tubulin was used as loading control.Tumour lysates from PyVmT/WT and PyVmT/Usp18 KO mice were analysed for p-Stat1, total Stat1 and Cxcl10 by Western blotting. Tubulin was used as loading control.Knockdown efficiency of PyVmT/Usp18 KO MECs stably expressing IL-28R1 shRNA was analyzed by IL-28R1 Western blotting. Tubulin was used as loading control.PyVmT/Usp18 KO MECs transduced with control or IL-28R1 shRNA were injected into mammary fat pads of wild-type mice. Tumour growth was monitored weekly by measuring tumours with a calliper. Number of tumours analyzed: Control shRNA, *n* = 5; IL-28R1 shRNA, *n* = 5.If statistical significance was reached relevant *p* values are shown in the diagram.

Although the three Cxcr3 ligands were originally described as type II IFN (IFN-γ) inducible genes, they are also induced by type I IFN and TNF-α but preferences and strength of induction vary between the three (Groom & Luster, 2011). To investigate the molecular mechanism underlying Cxcl10 induction in breast cancer cells, we treated PyVmT/Usp18 WT MECs with TNF-α, IFN-β and IFN-λ to analyse whether these cells differentially express the Cxcr3 ligands in a cytokine-dependent manner. IFN-λs belong to the novel type III IFN family. The downstream signalling in this pathway is similar to type I IFN (IFN-α/β) and its specific receptor, which is different from type I IFN receptor, is mainly expressed on epithelial cells (Kotenko et al, 2003; Sommereyns et al, 2008). TNF-α treatment did not induce expression of any of the Cxcr3 ligands in PyVmT MECs as determined by qRT-PCR (Supporting Information Fig 4A). Intriguingly, induction of Cxcl10 and Cxcl11 was significantly higher upon IFN-λ treatment relative to IFN-β treated cells (Fig 6). Irf7 and Oas2 were included as reference type I IFN-inducible genes and showed lower induction in response to IFN-λ relative to IFN-β. This finding raised the possibility that increased Cxcl10 expression in PyVmT/Usp18 KO MECs may not just be due to hypersensitivity to type I IFN signalling but rather due to loss of an inhibitory effect of Usp18 on type III IFN signalling. To test this hypothesis we treated vector control and Usp18 rescued PyVmT/Usp18 KO MECs with IFN-λ for 30 h and determined transcript levels of the same genes analyzed in Fig 6 by qRT-PCR. Relative to untreated cells most of the genes showed a statistically significant decrease in transcription when Usp18 was present, suggesting an inhibitory effect of Usp18 on type III IFN signalling (Fig 6). If Usp18 interferes with type III IFN signalling at an early point in the signalling cascade, similar to its inhibitory effect on type I IFN signalling, reduced levels of phosphorylated Stat1 should be observed in the presence of Usp18 upon IFN-λ treatment. Indeed, induction of IFN-λ signalling in rescued PyVmT/Usp18 KO MECs led to reduced levels of phosphorylated Stat1 (Fig 6). Interestingly, the inhibitory effect of Usp18 on phosphorylation of Stat1 was more significant upon IFN-λ treatment than IFN-β treatment. The human epithelial cell line ARPE19 stably expressing USP18 showed the same negative effect on STAT1 activation when treated with IFN-λ, suggesting a conserved regulatory mechanism across species (Supporting Information Fig 4B). In order to correlate elevated levels of phosphorylated Stat1 (p-Stat1) with increased Cxcl10 expression in our tumour model we probed tumour lysates from PyVmT/Usp18 WT and PyVmT/Usp18 KO mice for p-Stat1 and Cxcl10. A correlation between elevated phosphorylation of Stat1 and Cxcl10 was observed in tumour lysates from Usp18 KO mice (Fig 6). To further examine the specific role of IFN-λ signalling in our model we generated IL-28R1 knockdown PyVmT/Usp18 KO MECs for use in tumour cell injection experiments. Out of three different shRNAs directed against IL-28R1 only one showed reasonable reduction of IL-28R1 protein levels of about 70% (Fig 6) and was then used in subsequent experiments. Mice injected with PyVmT/Usp18 KO IL-28R1 shRNA MECs showed dramatically increased tumour growth compared to control shRNAPyVmT/Usp18 KO MECs (Fig 6). This finding suggests that hypersensitivity to IFN-λ in PyVmT/Usp18 KO MECs is of significant importance for inhibition of tumour progression.

## DISCUSSION

IFNs, induced during tumour development, are believed to play a key part in the early elimination phase of immunoediting (Matzinger, 1994). Throughout this phase immune cells locate, recognize and destroy developing tumours. Since PyVmT/Usp18 deficient cells are hypersensitive to type I IFN and, as we report here, type III IFN, delayed tumour onset in PyVmT/Usp18 KO mice is expected. However, the lack of Usp18 did not lead to a significant increase in tumour latency when compared to PyVmT/Usp18 WT mice (Fig 1). This can be explained by the aggressiveness of PyVmT driven mammary tumourigenesis in the FVB background, which may make it difficult to detect a significant difference at this early stage of tumour development (Davie et al, 2007). Accordingly, the inhibitory effect of Usp18 deficiency on tumour progression became more apparent over the period of study which is likely due to the gradual establishment of an antitumour cytokine environment (Fig 5).

Using animal models for different cancers including myeloma, neuroblastoma, melanoma, colon or breast cancer, several groups have reported a tumour-suppressive effect of Cxcl10 (Haabeth et al, 2011; Mohty et al, 2010; Pertl et al, 2001; Tominaga et al, 2007; Wang et al, 2010). Cxcl10 has strong angiostatic activity (Luster et al, 1995) and acts as a chemoattractant for Th1 subtype T cells (Bonecchi et al, 1998). Mammary tumours of mice and cultured PyVmT MECs lacking Usp18 had elevated levels of Cxcl10 (Fig 4). Consequently, tumours of PyVmT/Usp18 deficient mice showed reduced angiogenesis (Fig 2), increased infiltration of CD4^+^ T cells (Fig 3) and a Th1/M1-biased cytokine profile (Fig 4). Altogether these data suggest a prominent role for Cxcl10 in the observed phenotype of PyVmT/Usp18 KO mice. Tumour injection experiments using PyVmT/Usp18 deficient MECs with reduced Cxcl10 expression further underlined the importance of Cxcl10 produced by PyVmT MECs for inhibiting tumour progression in the absence of Usp18 (Fig 5). However, we cannot exclude that Usp18 deficient macrophages, T cells or other stromal cells also contribute to the observed Th1/M1 bias by secreting related cytokines. The reduction in lung metastasis (Fig 2), however, based on our current knowledge, is not linked to Cxcl10 and is likely a result of reduced invasiveness of Usp18 KO MECs (Fig 2) and a general increase of basal expression of IFN genes in Usp18 KO mice (Reid et al, 1981). Furthermore, the importance of CD4^+^ T cells for the observed phenotype, as demonstrated in MEC injection experiments with depleted CD4^+^ T cells (Fig 3), is most likely linked to the upregulation of Cxcl10 in PyVmT/Usp18 KO tumours. The general role of CD4^+^ T cells in tumour progression, however, is still controversial. Depending on the applied tumour cells and the used tumour model available data varies from complete rejection to enhanced tumour progression in the absence of CD4^+^ T (Jing et al, 2009; Kmieciak et al, 2011; Yu et al, 2005). This demonstrates, that the cytokine milieu specific to tumour cells and tumour stroma has a dramatic effect on the impact of CD4^+^ T cells on tumour progression, which is in accordance with our findings. In our mammary tumour model we show that Usp18 deficiency can reverse the effect of CD4^+^ T cells on tumour growth. This interesting finding presents a novel mechanism by which the immune system can be regulated during cancer progression.

**Figure 7:**
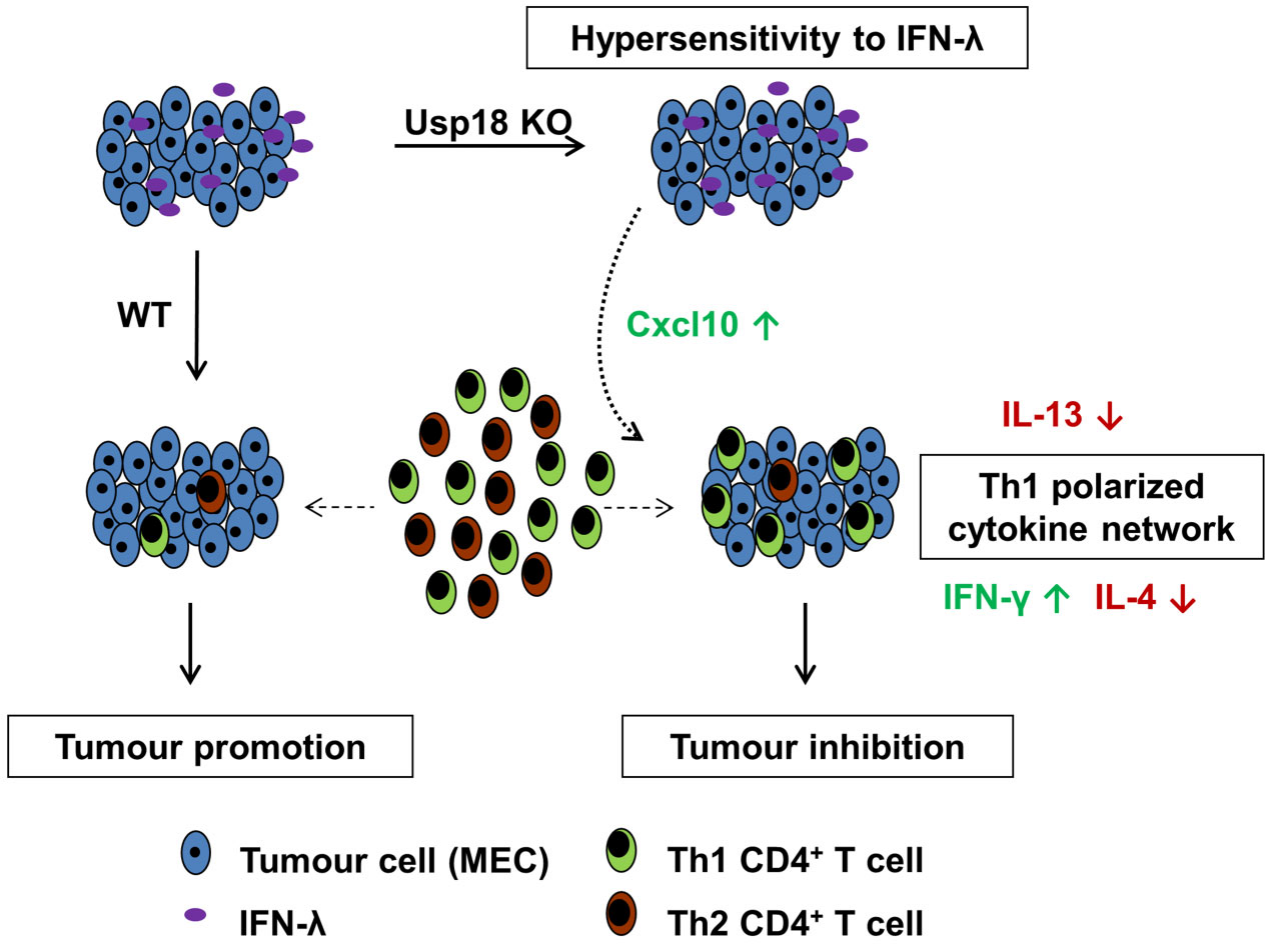
A model demonstrating the potential mechanisms by which Usp18 deficient MECs create a tumour-suppressive microenvironment in response to IFN-λ In contrast to WT MECs, Usp18 KO MECs exposed to IFN-λs in the tumour microenvironment dramatically upregulate secretion of Cxcl10. High levels of Cxcl10 (and other Cxcr3 ligands) then attract Th1-polarized CD4^+^ T cells to the tumour site establishing a tumour-suppressive Th1-biased cytokine network.

IFN-λs are generally believed to be weaker inducers of IFN gene expression than IFN-α/βs (Doyle et al, 2006). In our system, however, upregulation of both Cxcl10 and Cxcl11 in PyVmT MECs was more sensitive to IFN-λ than to IFN-β treatment, whereas Irf7 and Oas2 showed weaker induction in response to IFN-λ (Fig 6). Induction of Irf7 and Oas2 expression by IFN-λ was reduced by approximately 50% as compared to IFN-β, which is in accordance with the average difference reported by Doyle et al comparing gene expression profiles of HepG2 cells upon IFN-λ or IFN-α treatment (Doyle et al, 2006). In contrast, a different report showed that treatment of a murine melanoma cell line with IFN-λ led to a stronger induction of MHC class I expression than IFN-α (Lasfar et al, 2006). This corroborates our observation that IFN-λ may be more potent than type I IFNs in a gene and cell-type specific manner. In our model, induction of the Cxcr3 ligands in PyVmT MECs is more sensitive to IFN-λ than IFN-β, which may also be dependent on the cytokine concentrations present in the tumour microenvironment or used in our studies. Thus, we see higher levels of these chemokines in both PyVmT/Usp18 KO tumours and in *in vitro* cultured PyVmT MECs as compared to the “reference” IFN-inducible genes Irf7 and Oas2 (Fig 4). Moreover, our data show that Cxcl10 is the Cxcr3 ligand with the highest relative induction upon IFN-λ treatment (Fig 6), which is consistent with the observed highest relative upregulation of Cxcl10 inPyVmT/Usp18 deficient tumours and MECs (Fig 4). The preference of the Cxcr3 ligands for IFN-λ over IFN-β, together with the newly identifiedhypersensitivityofPyVmT/Usp18 deficient cells to IFN-λ can explain the high levels of Cxcl10, which are likely to play a crucial role in the establishment of the observed Th1-biased antitumour environment. Interestingly, in a murine model for allergic asthma, overexpression or administration of IFN-λ promoted Th1-cell differentiation while suppressing Th2-mediated responses (Koltsida et al, 2011). This corroborates our findings and suggests a role for IFN-λ that goes beyond the antiviral immune response. Figure 7 is a simplified model outlining the importance of IFN-λ hypersensitivity and the presence of CD4^+^ T cells in tumour inhibition in the absence of Usp18. In accordance with our working model, the tumour-suppressive effect of Usp18 KO MECs was abolished when sensitivity to IFN-λ was reduced (Fig 6) or Usp18 KO MECs were injected into CD4^+^ T-cell-depleted mice (Fig 3).

The source of IFN-λ in our model is not known but similar to type I IFNs IFN-λ can be produced by a variety of cells with myeloid-derived DCs (MD-DCs) and plasmacytoid DCs (pDCs) being the most important sources (Coccia et al, 2004; Siren et al, 2005). It was also shown that a number of cancer cell lines produce significant levels of IFN-λ (Kotenko et al, 2003; Tissari et al, 2005). Currently we do not know the exact molecular mechanism as to how Usp18 interferes with IFN-λ induced Jak/Stat signalling. Since we have shown that binding of Usp18 to type I IFN receptor subunit 2 (Ifnar2) is important for Usp18 mediated downregulation of type I IFN signalling (Malakhova et al, 2006), we investigated a potential interaction of Usp18 with the IFN-λ specific receptor subunit IL-28R1. This receptor subunit shows sequence and structural similarity to Ifnar2 in its cytoplasmic domain. However, the performed co-immunoprecipitation experiments did not suggest direct interaction of USP18 with IL-28R1 (Supporting Information Fig 5). Therefore, the molecular mechanism of Usp18-mediated inhibition of type III IFN signalling requires further study. Remarkably, we found that Usp18 KO MECs with reduced levels of IL-28R1 exhibit more aggressive tumour development which points to a novel protective role of IFN-λ signalling in tumour development of epithelial cancers, specifically in the absence of Usp18 (Fig 6).

Since Usp18 is the Isg15 specific deconjugating enzyme (Malakhov et al, 2002) it is possible that enhanced conjugation of Isg15 to target proteins (ISGylation) in PyVmT/Usp18 deficient cells also affects tumourigenesis. Indeed, a recent report showed that doxorubicin-mediated tumour suppression in a tumour graft model requires protein ISGylation suggesting an important role of Isg15 conjugation in antitumour responses (Jeon et al, 2012). In our model, mammary tumours and *in vitro* cultured PyVmT MECs show significant levels of ISGylation, which are further elevated in the absence of Usp18 (Supporting Information Fig 6). Elevated levels of protein ISGylation in MECs or the tumour stroma do not seem to have a major impact on tumour development in our orthotopic tumour cell injection model (Fig 5). We did, however, observe significantly reduced tumour growth at early time points when injecting WT MECs into Usp18 KO mice that was then lost over the course of the study (Fig 5). This interesting finding suggests that Usp18 deficient stromal cells with increased expression levels of IFN genes and elevated protein ISGylation may mediate an inhibitory effect on tumour onset upon injection of MECs but do not affect tumour growth rate. Analysis of the effect of protein ISGylation on tumour initiation and progression in the PyVmT mouse model exceeds the scope of this report but is currently being investigated in our laboratory. Interestingly, initial analysis of PyVmT mice lacking Ube1l, the E1 enzyme for Isg15 conjugation, suggests a protective role of protein ISGylation against mammary tumour growth (unpublished observations).

In summary, we show that loss of Usp18 has tumour-suppressive activity in a spontaneous mammary cancer model. Lack of Usp18 modulates the tumour microenvironment by upregulating Cxcr3 ligands in PyVmT MECs, particularly Cxcl10, creating aTh1-biased cytokine network. Remarkably, the generation of this antitumour environment in PyVmT/Usp18 KO mice does not seem to be orchestrated by a general upregulation of IFN genes but rather a specific subset that appears to be more sensitive to type III IFN stimulation. Furthermore, we identified Usp18 as a regulator of CD4^+^ T-cell function in tumour protection. Thus, targeting Usp18 may be a viable approach to overcome relapse or enhance immunotherapeutic approaches, such as adoptive T-cell transfer. The E3 ubiquitin ligase Cbl-b, for instance, was identified as a key regulator of spontaneous antitumour activity of cytotoxic T cells and a Cbl-b based drug for immunotherapy is currently under development (Chiang et al, 2007; Loeser et al, 2007). Usp18 as a therapeutic target may provide advantages in combination with IFN therapy, especially IFN-λ. In the treatment of epithelial cancers a specific Usp18 inhibitor in combination with IFN-λ administration could significantly boost the antitumour immune response and reduce side effects due to IFN-λ's targeted activity on epithelial cells. In fact, a reduction in side effects was observed in a clinical trial testing efficacy of PEG-IFN-λ compared to PEG-IFNα in the treatment of hepatitis C (Miller et al, 2009). Moreover, two recent reports underline the importance of (a) IFN signalling for suppression of breast cancer metastasis and (b) protein ISGylation for antitumour responses (Bidwell et al, 2012; Jeon et al, 2012). Considering that Usp18 regulates both these pathways, the mentioned reports together with our findings, strongly support the concept that Usp18 is a highly interesting and promising drug target in breast cancer and possibly other solid tumours. Therefore, it is reasonable to speculate that a drug targeting both the IFN inhibitory function and enzymatic activity of Usp18 would represent the most potent way to exploit its function for cancer therapy.

## MATERIALS AND METHODS

### Mice and tumour monitoring

Usp18 deficient mice in the FVB background were described previously (Cong et al, 2012). Transgenic FVB mice expressing PyVmT under the control of the mouse MMTV-LTR were crossed with wild-type or Usp18 deficient mice. Female mice heterozygous for the PyVmT transgene and homozygous null or homozygous wild-type for Usp18 were used in these studies. From 3 weeks onwards female mice were palpated twice weekly to monitor the development of mammary tumours. Tumour bearing mice were sacrificed at 13 weeks of age for analysis. For metastasis studies, mouse lungs were harvested at 13 weeks of age and the number of lung metastases was determined by H&E staining of 10 serial lung sections per mouse. In tumour cell injection experiments, tumours were measured weekly in two dimensions with a calliper and diameters expressed as the mean of both measurements. All studies were carried out following the NIH guidelines for the care and treatment of experimental laboratory rodents.

### Plasmids, reagents and antibodies

Full-length murine Usp18 with N-terminally added HA-tag sequence was amplified from cDNA of IFN-α treated RAW Macrophages and cloned into pMSCV-puro (Clontech). pMSCV-puro-hUSP18 and pCDNA3.1-USP18 were previously described (Burkart et al, 2012). Mammalian expression constructs coding for the intracellular domain of IFNAR2 or IL-28R1in the pcDEF3 vector were kindly provided by Dr. Sergei Kotenko. Antibodies against β-tubulin (Sigma), HA (Covance), Stat1, p-Stat1, anti-Caspase 3 (all Cell Signal), Cxcl10, IL-28R1 (Santa Cruz), CD31, CD4 (both BD Pharmingen), CD4-PerCP-Cy5.5, CD4-R-phycoerythrin, CD326-R-phycoerythrin, CD25-PerCP-Cy5.5 (all ebioscience) and CD8-R-Phycoerythrin (Invitrogen) were purchased as indicated. Anti-CD4 antibody for depletion experiments was from Biolegend. Anti-Isg15 and anti-USP18 antibody was previously described (Malakhov et al, 2002, 2006). Lentiviral murine Cxcl10 shRNA set was purchased from Open Biosystems and lentiviral murine IL-28R1 shRNA set was from Origene. Recombinant human IFN-λ1 was purchased from Peprotech and recombinant murine IFN-β was from EMD Millipore.

### Immunohistochemical and immunofluorescent staining

Tumour samples were either fixed in 4% paraformaldehayde, embedded in paraffin and sectioned or embedded into OCT compound (Tissue-Tek) and frozen in liquid nitrogen. For histochemical analysis paraffin sections were stained with H&E according to standard protocols. Immunostainings were performed on paraffin or frozen sections using VECTASTAIN ABC peroxidase/DAB staining kits (Vector Laboratories). For immunofluorescent staining, frozen sections were prepared at 8 µm thickness and stained with antibodies against CD4 followed by FITC conjugated secondary antibodies. Cell nuclei were counterstained by 49,69-diamino-2-phenylindole (DAPI). Images were captured using an Olympus camera model DP71 on a Olympus BX51 microscope at 100× or 200× magnification.

**The paper explained**

PROBLEM:

While the understanding of the role of the immune system in cancer progression has made significant progress over the past decades, the successes of cancer immunotherapies have been inconsistent. For instance, in the case of anticancer vaccines only a minority of patients actually benefit from vaccine-induced T cells, which is likely due to inefficient homing of tumour-specific T cells to the tumour site. We hypothesize that new therapies that can efficiently attract tumour-suppressive immune cells to the tumour area will improve efficacy of current cancer therapies.

RESULTS:

We report that Usp18 deficient MECs secrete elevated levels of the T-cell chemoattractant Cxcl10. Accumulation of this chemokine leads to enhanced recruitment of Th1 cells and generation of a tumour-suppressive cytokine environment resulting in inhibition of mammary tumour growth. Furthermore, we show that Usp18 is a novel negative regulator of IFN-λ signalling, which promotes upregulation of Cxcl10 in MECs lacking Usp18.

IMPACT:We identified Usp18 as a regulator of the tumour microenvironment and promising candidate for breast cancer immunotherapy. Since IFN-λ acts exclusively on epithelial cells, a combined treatment with a Usp18 inhibitor will locally boost the antitumour immune response with minimal off-target effects. This combination of a Usp18 specific inhibitor with IFN-λ treatment may be of great benefit for the treatment of epithelial cancers.

### Western blotting and sandwich ELISA

Protein extracts were prepared from mammary tumours, isolated tumour cells or established cell lines by lysis in modified RIPA buffer as described previously (Burkart et al, 2012). Lysates were then fractionated by SDS–PAGE electrophoresis, transferred to nitrocellulose membranes, reacted with primary and fluorophore-conjugated secondary antibodies, and signals detected with the Odyssey system (LI-COR). For Cxcl10 ELISA, MECs were seeded onto a 6-well plate, supernatants were collected after 48 h, spun down to remove debris and immediately analyzed according to manufacturer's protocol (R&D Systems).

### Isolation of mammary tumour cells for flow cytometric analysis and establishment of primary cell lines

Single cell suspensions were established from spontaneous mammary tumours arising in FVB MMTV-PyVmT females. Tumours were minced and incubated at 37°C for 3 h in 5 ml of Ham's F12K medium containing 1 mg/ml collagenase (Roche), 2 mg/ml soybean trypsin inhibitor (Sigma) and 2% BSA. After addition of FCS-containing medium, the suspension was passed through a 70 µm nylon filter (Fisher Scientific) and red blood cells removed with ACK lysing buffer. Single cells were pelleted by centrifugation and resuspended in PBS for immediate flow cytometric analysis. Stained cells were analyzed by a FACSCanto cytometer (BD Biosciences). For establishment of MEC cell lines cells were cultured in DMEM:F12 (1:1) medium mix containing 5% FCS, 2.5 µg/ml amphotericin B, 10 µg/ml penicillin–streptomycin and MITO+ (BD Biosciences). Non-epithelial cells were removed by differential passaging and epithelial origin of established cell lines confirmed by CD326 staining after five passages (CD326^+^ > 98%).

### Orthotopic injections into the mammary fat pad

*In vitro* cultured MECs (1 × 10^6^) resuspended in 1 mg/ml matrigel solution were injected into the abdominal mammary fat pads of female mice. Growing tumours were monitored once a week with a calliper.

### Cell growth assays

Cells (5 × 10^3^) were seeded in each well of a 96-well plate on day 0. Cell growth was monitored for 1 week with CellTiter 96® AQueous One Solution Reagent according to manufacturer's instructions (Promega).

### Matrigel invasion assays

MEC invasion was tested using a BD BioCoat Matrigel Invasion Chamber (BD Biosciences). The cells were suspended in serum-free DMEM and then added to the upper chamber at a density of 3 × 10^5^ cells/well. The bottom chamber contained DMEM supplemented with 5% FBS as chemoattractant. Cell invasion into the Matrigel was determined after 24 h of culture at 37 °C. The membrane containing invading cells was fixed using methanol, stained with Wright-Giemsa, and invasion was quantified by light microscopy after removing the non-invading cells on the upper side of the membrane with cotton swabs.

### RNA isolation and qRT-PCR analysis

Total RNA from tumour tissue or MECs was isolated using RNeasy (Qiagen) according to the manufacturer's instructions. For qRT-PCR analyses, equal amounts of RNA were reverse-transcribed by qScript (Quanta Biosciences) and the resulting cDNA templates were subjected to qRT-PCR using SYBR Green detection system on the CFX96 thermal cycler (BIO-RAD). Primer sequences are available upon request.

### Statistical analysis

Statistical significance was evaluated by a paired *T*-test, using *p* < 0.05 as indicative of statistical significance. Kaplan–Meier tumour-free survival data were compared using the log-rank test.

## Supplementary material

As a service to our authors and readers, this journal provides supporting information supplied by the authors. Such materials are peer reviewed and may be re-organized for online delivery, but are not copy-edited or typeset. Technical support issues arising from supporting information (other than missing files) should be addressed to the authors.
