# Supplementary material for: Usp18 deficient mammary epithelial cells create an antitumour environment driven by hypersensitivity to IFN-λ and elevated secretion of Cxcl10
Source: EMBO Mol Med. 2013 May 16;5(7):967–82. doi: 10.1002/emmm.201201864 (PMC3721472; doi:10.1002/emmm.201201864)
Supplement: Supplementary file 1 [file emmm0005-0967-SD1.pdf]

EMBO Molecular Medicine

Usp18 deficient mammary epithelial cells create an antitumour environment driven by hypersensitivity to IFN-λ and elevated secretion of Cxcl10

Christoph Burkart, Kei-ichiro Arimoto, Tingdong Tang, Xiuli Cong, Nengming Xiao, Yun-Cai Liu, Sergei V. Kotenko, Lesley G. Ellies and Dong-Er Zhang

Corresponding author: Dong-Er Zhang, University of California, San Diego

Review timeline:

Submission date:	24 August 2012
Editorial Decision:	10 October 2013
Revision received:	03 March 2013
Editorial Decision:	26 March 2013
Revision received:	04 April 2013
Accepted:	05 April 2013

Transaction Report:

(Note: With the exception of the correction of typographical or spelling errors that could be a source of ambiguity, letters and reports are not edited. The original formatting of letters and referee reports may not be reflected in this compilation.)

Editor: Natascha Bushati / Céline Carret

1st Editorial Decision 10 October 2013

Thank you for the submission of your manuscript "Usp18 deficient mammary epithelial cells create an antitumour environment by elevated secretion of Cxcl10" to EMBO Molecular Medicine. We have now heard back from the three referees whose comments are below. You will see that they consider your manuscript to be potentially of rather significant interest. However, they also raise a number of concerns about the study, which would have to be addressed in a substantive revision of the manuscript.

Importantly, reviewers #2 and #3 highlight that additional in vivo data addressing the Usp18-dependent role of CD4+ T cells in mediating the antitumour environment are required to strengthen the study. These reviewers also require more careful characterisation of the T cell immunophenotype in the presented tumours. In addition, they would like to see further analyses of the tumour phenotypes (WT; Usp18-KO; Usp18-KO, CXCl10 shRNA) in the form of careful staging and histochemistry.

Reviewer #1 notes that the mechanistic link between IFN-gamma and the Usp18-KO phenotype should be strengthened experimentally and suggests to test whether knockdown of IL28-R1 in Usp18-KO MECs prior to transplantation affects tumour growth. In addition, this reviewer suggests investigating the effect of catalytically inactive Usp18 on in vivo tumour growth. We agree that the

two latter experiments would strengthen the manuscript, but do not consider this as a precondition for the re-review of a resubmission. Finally, we do not consider the suggestion of reviewer #3 to investigate the effects of CXCL10 overexpression as essential for re-review of a resubmission.

In our view the suggested revisions would render the manuscript much more compelling and interesting to a broad readership. We therefore hope that you will be prepared to undertake the recommended experimental revision.

Revised manuscripts should be submitted within three months of a request for revision. If your revision will have to exceed this time frame, please contact the editor. Please also contact the editor as soon as possible if similar work is published elsewhere.

I look forward to seeing a revised form of your manuscript in due course.

***** Reviewer's comments *****

Referee #1 (Comments on Novelty/Model System):

Mouse models are appropriate for tumorigenesis and anti-tumor immunity studies

Referee #1 (General Remarks):

This work focuses on the role of Usp18 gene that function as an enzyme to dis-assemble ISG15 chains and an inhibitor of IFN pathways in regulating the conditions of tumor microenvironment (TME) that control progression of breast carcinomas.
Using the PyVmT-based mammary tumorigenesis model, they demonstrate that knockout of Usp18 suppresses tumor growth and progression. Studies using transplantation of mammary epithelial cells (MEC) further suggest a cell-autonomous role of Usp18 and point to elevated Cxcl10 secretion and ensuing recruitment of cytotoxic T cells as an underlying mechanism. Given that Usp18-dependent phenotype correlates with responsiveness to IFN-lambda, authors implicate these cytokines in controlling the process.
Overall, this work is very important for our understanding of the mechanisms that regulate anti-tumor immunity. There is also a clear translational relevance: if Usp18 acts via an enzymatically-dependent mechanism, potential inhibition of this enzyme may prevent tumor-imposed immunoediting and boost the ability of a host to combat breast cancer development and progression. Most of experiments are very well designed, results are compelling and support the conclusions made.

The following suggestions may help to further strengthen the manuscript:

Major points:

1. The mechanistic link to IFN-lambda is largely based on correlative data. Will knockdown of IL28-R1 in MEC from Usp18-null animals prior to transplantation affect the tumor growth?
2. A suggestion that inhibition of Usp18 might help the host to reject tumors hinges on the assumption that inhibition phenotype will be equivalent to the knockout. An experiment comparing in vivo growth of Usp18-null MEC MEC re-constituted with catalytically inactive Usp18 (versus wild type protein) would greatly strengthen such proposition and foster the clinical relevance of this study.

© EMBO

Minor points:

1. Fig. 2C: a positive control for cleaved caspase 3 will help the readers to better understand significance of this data
2. Fig.2E legend states the use of frozen sections whereas the Methods chapter describes that tissues were fixed in paraformaldehyde: which one is correct?
3. Fig. 2G could be better described in the text/Figure legend
4. If possible, authors should keep consistent labeling for knockout cells (open bars?) throughout Fig. 4A
5. It appears that there is a statistically significant effect of Usp18-null stroma at early time points (Fig. 5A). This finding might be important and should be discussed
6. All experiments that use MEC will benefit from demonstrating the levels of reconstituted Usp18 in comparison with that in wild type cells. This data could be added to Fig.6C

Referee #2 (Comments on Novelty/Model System):

This is an interesting manuscript, which reports potentially novel data.

Referee #2 (General Remarks):

In the manuscript entitled "Usp18 deficient mammary epithelial cells create an antitumour environment by elevated secretion of Cxcl10", Burkart and co-authors analyze the role of Usp18, a negative regulator of the interferon response, in a mouse model of breast cancer. By comparing wild-type (WT) with Usp18 knockout (Usp18-KO) polyomavirus middle-T (PyVmT) transgenic mice, the authors show that the absence of Usp18 correlates with a less aggressive tumor progression (decreased tumor burden; lower vascular density; lover incidence of invasive carcinomas and metastasis). Decreased tumor burden in Usp18-KO mice was not due to changes in the rates of cancer cell proliferation or apoptosis, but was associated with higher tumor infiltration by Th1 polarized CD4+ T cells. By employing elegant and compelling tumor transplant experiments, the authors show that tumor inhibition in Usp18-KO mice was due to cell-autonomous effects in the cancer cells (and not in the tumor-associated stromal cells). They found that absence of Usp18 in the cancer cells upregulated expression and secretion of the cytokine, CXCL10, which is a potent chemoattractant for T cells. Based on this, Burkart and colleagues speculate that Usp18 deficiency in the cancer cells increases CXCL10 secretion and recruitment of CD4+ lymphocytes into the tumor, possibly establishing a tumor-suppressive microenvironment. In agreement with this hypothesis, PyVmT Usp18-KO cancer cells depleted of CXCL10 by means of a shRNA against Cxcl10 display a "rescued" tumor growth phenotype (compared with the CXCL10-proficient Usp18-KO cancer cells) when transplanted in WT recipient mice. The authors further show that absence of Usp18 increases mammary cancer cell sensitivity to the type III interferon, IFN-lambda, leading to higher production of CXCL10, possibly in a STAT1-dependent manner. Interestingly, it was found that IFN-lambda is a stronger inducer of CXCL10 than the type I IFN, IFN-alpha.

This manuscript provides interesting data and new insight into the putative role of the interferon-inducible gene, Usp18, in mammary carcinogenesis. The authors describe a potentially novel mechanism of Usp18 modulation of tumor-protective immunity that involves CXCL10 induction by IFN-lambda; only recently, it has been shown that Usp18 modulates conventional dentritic cell development via its inhibitory effects on type I IFN signaling. The manuscript is well written and the data are clearly presented. I have a few comments and suggestions that should improve the quality and robustness of this original contribution.

© EMBO

Main comments and suggestions:

1) The authors should reproduce some of their findings (e.g., the transplant experiments in Figure 5A-B) using wild-type (WT) cancer cells. Indeed, it is not clear why the authors only employed Usp18 "rescued" cells obtained by retroviral transduction of Usp18-KO cells. How can they exclude that they are indeed overexpressing Usp18 to supra-physiological levels? What is the vector copy number in the transduced cells?
2) Most of the data in Figures 1-3 are descriptive of gross tumor phenotypes. Because tumorigenesis is delayed in Usp18-KO mice, it is very likely that the observed phenotypes (vascular density, metastatic colonization, immune cell infiltration, Th1/Th2 polarization of immune cells) mostly reflect the overall different stage of tumor progression in WT vs. Usp18-KO mice. To rule out this concern, the authors should compare stage-matched (or at least size-matched) tumors, e.g., by analyzing WT and Usp18-KO tumors at different time-points.
3) The authors state that slower tumor development in Usp18-KO mice is caused by enhanced CXCL10-driven CD4+ T cell recruitment to the tumors, which in turn enhances antitumor immunity. However, this hypothesis is not fully supported by the data presented. Burkart et al. should demonstrate that the CD4+ T cells play a protective role in tumors in a Usp18-dependent manner. The authors could address this important issue by transiently depleting CD4+ T cells using blocking antibodies, or by performing transplant experiments in RAG-/- mice.

Other questions:
4) Does IFN-lambda upregulate Usp18 in mammary carcinoma cells?
5) What is the immunophenotype of the CD4+ T cells in Usp18-KO vs. WT tumors? Do they have a more pronounced activated phenotype? Finally, what is the status of CD8+ (cytotoxic) and regulatory T cell infiltration in the tumors? Better characterization of the T cell immunophenotype in the tumors may strengthen the authors' hypothesis that Usp18 deletion in the cancer cells unleashes tumor-protective immunity.

Referee #3 (Comments on Novelty/Model System):

pls see review comments

Referee #3 (General Remarks):

The manuscript by Burkart et. al., shows that ablation of USP18 in mammary epithelial cells generates an antitumorigenic microenvironment resulting in reduced tumor burden in the PyVMT mammary carcinoma model. The observations made in the paper are intriguing and are suggestive of activation of a Th1 response in USP18 KO tumors. The manuscript is well written and is able to convey the significance of the study. However, there are several aspects in the paper, which require further clarification in order to validate and strengthen the conclusions made in this manuscript.

Minor comments:

Figure 2G should include PyVMT WT MECs also in the invasion assay.

Fig 2E requires quantitation and power calculations.

Fig. 3E should be instead included in fig.1

Fig. 4B should indicate the duration of MEC culture to generate supernatant.

Fig. 6C should be quantitated for phosphorylated STAT1 with reference to tubulin as well as

STAT1. The authors should discuss the source of IFN- in the tumor microenvironment as they claim it to be the most sensitive pathway for USP18 ablation. If this is indeed true, then cellular identification of the source is required.

Fig 6B CXCL10 levels are inconsistent with that shown in Fig 4A

Major comments:

Fig. 1 does not show if there is overall survival differences in USP18 KO mice as compared to WT PyVMT. This is required.

Fig. 2 touches upon the mechanism of reduced tumor burden in USP18 KO mice. However, the assays are inappropriate. Histochemical evaluation of primary tumor sections is required to evaluate changes in proliferation and/or cell death. Invasion assays and metastasis rate is neither informative nor helpful in this context. Differences in vessel density would be better supported by analyzing the expression levels of angiogenesis regulators in tumors.

Fig.3 should include additional cell surface markers to strengthen data shown, e.g., CD11b, Ly6C, and Ly6G add resolution to the myeloid cell repertoire, which is not represented comprehensively by F4/80. Authors concluded CD4 T cells as the effector cell population based on their increased representation in cellular composition of USP18 KO tumors. CD4 T cells may play a critical role in USP18 KO tumors but this requires additional analyses to support, including depletion of all CD4+ T cells as opposed to mere depletion of T reg cells. In addition, IF studies to examine the FoxP3 and CD25 status of CD4 T cells would be helpful.. Mere quantitation of CD8 T cells is not be conclusive regarding their influence on tumor growth. These must be evaluated IFN- production, granzyme B, CD25 and proliferation.

Fig. 5 shows that CXCL10 is a MEC-derived paracrine regulator of the tumor microenvironment. This data would be improved by analysis of vessel density, proliferation and apoptosis status in tumors derived from USP18 KO MECs with CXCl10 knockdown. Moreover, tumor cell composition should also be analyzed in these tumors with a focus on CD4 T cells. It is obvious to inquire here if recombinant CXCl10 administration in WT-PYMT mice or ectopic expression of CXCL10 in MECs will cause stunted tumor growth. Moreover, since CXCL10 is an angiostatic chemokine, ex vivo endothelial invasion assays should be considered.

Fig 6 shows inhibition of STAT1 activation by USP18 expression and increased CXCL10 production when IFN is applied to USP18 KO MECs. There is no data provided to indicate that these changes are also observed in vivo in the tumors. Tumor sections should be quatitatively evaluated for CXCL10 and pSTAT1 presence and co-localization. Lack of this data necessitates removal of Fig 6 and mere mention of it as a possible scenario in the conclusion.

1st Revision - authors' response 03 March 2013

We thank all of the reviewers for their positive, valuable, and constructive comments about our manuscript. We have addressed these comments accordingly. Additions and changes to the manuscript are in blue colored font in the text.

Here are the point-to-point responses:

Referee #1:

This work focuses on the role of Usp18 gene that function as an enzyme to dis-assemble ISG15 chains and an inhibitor of IFN pathways in regulating the conditions of tumor microenvironment (TME) that control progression of breast carcinomas.
Using the PyVmT-based mammary tumorigenesis model, they demonstrate that knockout of Usp18 suppresses tumor growth and progression. Studies using transplantation of mammary epithelial cells (MEC) further suggest a cell-autonomous role of Usp18 and point to elevated Cxcl10 secretion and ensuing recruitment of cytotoxic T cells as an underlying mechanism. Given that Usp18-dependent phenotype correlates with responsiveness to IFN-lambda, authors implicate these cytokines in controlling the process.
Overall, this work is very important for our understanding of the mechanisms that regulate anti-tumor immunity. There is also a clear translational relevance: if Usp18 acts via an enzymatically-dependent mechanism, potential inhibition of this enzyme may prevent tumor-imposed immunoediting and boost the ability of a host to combat breast cancer development and progression. Most of experiments are very well designed, results are compelling and support the conclusions made.
The following suggestions may help to further strengthen the manuscript:

Major points:
1. The mechanistic link to IFN-lambda is largely based on correlative data. Will knockdown of IL28-R1 in MEC from Usp18-null animals prior to transplantation affect the tumor growth?

This is an excellent question. Using three different shRNA clones we generated Usp18 KO MECs stably expressing IL-28R1 shRNA. Only one shRNA showed good reduction of IL-28R1 protein levels (Fig 6E) and was used in subsequent transplantation experiments (Fig 6F). Mice injected with PyVmT/Usp18 KO IL-28R1 shRNA MECs showed significantly enhanced tumour growth underlining the importance of IFN-λ signaling in the tumour suppressive phenotype of PyVmT/Usp18 KO MECs.

2. A suggestion that inhibition of Usp18 might help the host to reject tumors hinges on the assumption that inhibition phenotype will be equivalent to the knockout. An experiment comparing in vivo growth of Usp18-null MEC re-constituted with catalytically inactive Usp18 (versus wild type protein) would greatly strengthen such proposition and foster the clinical relevance of this study.

We added data from additional transplantation experiments comparing growth of Usp18 KO+Usp18 and Usp18 KO+Usp18-C61S MECs to figure 5B. Our results show that in this experimental setting both cell lines lead to similar tumour development indicating that the inhibitory effect of Usp18 on IFN signaling plays a more important role in this process.

Minor points:

1. Fig. 2C: a positive control for cleaved caspase 3 will help the readers to better understand significance of this data.

We replaced this figure with TUNEL assay performed on Usp18 KO and WT tumour sections.

2. Fig.2E legend states the use of frozen sections whereas the Methods chapter describes that tissues were fixed in paraformaldehyde: which one is correct?

Methods part corrected accordingly.

3. Fig. 2G could be better described in the text/Figure legend.

Text modified.

4. If possible, authors should keep consistent labeling for knockout cells (open bars?) throughout Fig. 4A

Labeling adjusted.

5. It appears that there is a statistically significant effect of Usp18-null stroma at early time points (Fig. 5A). This finding might be important and should be discussed.

We added a section in the discussion about this.

6. All experiments that use MEC will benefit from demonstrating the levels of reconstituted Usp18 in comparison with that in wild type cells. This data could be added to Fig.6C.

Requested panel added as Supporting Information Fig 2B.

<u>Referee #2:</u>

In the manuscript entitled "Usp18 deficient mammary epithelial cells create an antitumour environment by elevated secretion of Cxcl10", Burkart and co-authors analyze the role of Usp18, a negative regulator of the interferon response, in a mouse model of breast cancer. By comparing wild-type (WT) with Usp18 knockout (Usp18-KO) polyomavirus middle-T (PyVmT) transgenic mice, the authors show that the absence of Usp18 correlates with a less aggressive tumor progression (decreased tumor burden; lower vascular density; lover incidence of invasive carcinomas and metastasis). Decreased tumor burden in Usp18-KO mice was not due to changes in the rates of cancer cell proliferation or apoptosis, but was

associated with higher tumor infiltration by Th1 polarized CD4+ T cells. By employing elegant and compelling tumor transplant experiments, the authors show that tumor inhibition in Usp18-KO mice was due to cell-autonomous effects in the cancer cells (and not in the tumor-associated stromal cells). They found that absence of Usp18 in the cancer cells upregulated expression and secretion of the cytokine, CXCL10, which is a potent chemoattractant for T cells. Based on this, Burkart and colleagues speculate that Usp18 deficiency in the cancer cells increases CXCL10 secretion and recruitment of CD4+ lymphocytes into the tumor, possibly establishing a tumor-suppressive microenvironment. In agreement with this hypothesis, PyVmT Usp18-KO cancer cells depleted of CXCL10 by means of a shRNA against Cxcl10 display a "rescued" tumor growth phenotype (compared with the CXCL10-proficient Usp18-KO cancer cells) when transplanted in WT recipient mice. The authors further show that absence of Usp18 increases mammary cancer cell sensitivity to the type III interferon, IFN-lambda, leading to higher production of CXCL10, possibly in a STAT1-dependent manner. Interestingly, it was found that IFN-lambda is a stronger inducer of CXCL10 than the type I IFN, IFN-alpha.

This manuscript provides interesting data and new insight into the putative role of the interferon-inducible gene, Usp18, in mammary carcinogenesis. The authors describe a potentially novel mechanism of Usp18 modulation of tumor-protective immunity that involves CXCL10 induction by IFN-lambda; only recently, it has been shown that Usp18 modulates conventional dentritic cell development via its inhibitory effects on type I IFN signaling. The manuscript is well written and the data are clearly presented. I have a few comments and suggestions that should improve the quality and robustness of this original contribution.

Main comments and suggestions:

1) The authors should reproduce some of their findings (e.g., the transplant experiments in Figure 5A-B) using wild-type (WT) cancer cells. Indeed, it is not clear why the authors only employed Usp18 "rescued" cells obtained by retroviral transduction of Usp18-KO cells. How can they exclude that they are indeed overexpressing Usp18 to supra-physiological levels? What is the vector copy number in the transduced cells?

These are good points. We added a figure to the supplement showing results of transplantation experiments comparing tumour growth of WT MECs with Usp18 KO + Usp18 MECs (Sup Information Fig. 2C). Tumour growth between the two was not significantly changed over the time course of the study. In the initial manuscript we chose to only compare Usp18 KO MECs to Usp18 KO + Usp18 MECs derived from the same primary tumour in order to avoid Usp18 independent, secondary effects due to genetic heterogeneity of tumor cells derived from different tumours or mice. Furthermore, we added Western Blotting data showing protein levels of rescued HA-mUsp18 in comparison to endogenous Usp18 levels of untreated and IFN treated wildtype MECs (Supporting Information Fig 2B).

2) Most of the data in Figures 1-3 are descriptive of gross tumor phenotypes. Because tumorigenesis is delayed in Usp18-KO mice, it is very likely that the observed phenotypes (vascular density, metastatic colonization, immune cell infiltration, Th1/Th2 polarization of immune cells) mostly reflect the overall different stage of tumor progression in WT vs. Usp18-KO mice. To rule out this concern, the authors should compare stage-matched (or at least size-matched) tumors, e.g., by analyzing WT and Usp18-KO tumors at different time-points.

We agree with the reviewer’s concern that some of the features such as tumour morphology and vascular density could be related to delayed tumour progression in Usp18 KO mice. Unfortunately, within the limited time we were not able to fully address this question since we did not get the number of samples required for a conclusive study. However, increased production of Cxcl10 by Usp18 deficient MECs is maintained in isolated *in vitro* culture conditions, which suggests that enrichment of Th1 polarized T cells in the tumour microenvironment may persist throughout the different tumour stages. Regarding lung metastasis the observed difference is probably not solely due to delayed tumour growth since we also observed significant reduction in metastatic activity of Usp18 deficient MECs in *in vitro* assays (Fig 2G). The additional possibilities will be explored in future studies

3) The authors state that slower tumor development in Usp18-KO mice is caused by enhanced CXCL10-driven CD4+ T cell recruitment to the tumors, which in turn enhances antitumor immunity. However, this hypothesis is not fully supported by the data presented. Burkart et al. should demonstrate that the CD4+ T cells play a protective role in tumors in a Usp18-dependent manner. The authors could address this important issue by transiently depleting CD4+ T cells using blocking antibodies, or by performing transplant experiments in RAG-/- mice.

This is an excellent question. Studies with transient depletion of $CD4^+$ T cells using blocking antibodies strongly supported the role of $CD4^+$ T cells in Usp18 related tumour progression. The results are shown in new Fig 3D.

Other questions:

1) Does IFN-lambda upregulate Usp18 in mammary carcinoma cells?

Yes, as shown in Supporting Information Fig 2B.

2) What is the immunophenotype of the CD4+ T cells in Usp18-KO vs. WT tumors? Do they have a more pronounced activated phenotype? Finally, what is the status of CD8+ (cytotoxic) and regulatory T cell infiltration in the tumors? Better characterization of the T cell immunophenotype in the tumors may strengthen the authors' hypothesis that Usp18 deletion in the cancer cells unleashes tumor-protective immunity.

Since we observed elevated levels of Cxcl10 in both tumours and isolated MECs from Usp18 KO mice we characterized the cytokines present in tumours with regard to Th cell subtypes. Our analysis

showed a Th1/M1 cytokine bias within PyVmT/Usp18 KO tumours (Figure 4C/D). Furthermore, we did not detect a difference in Foxp3 levels between PyVmT/Usp18 KO and PyVmT/Usp18 WT tumours suggesting that generation of Tregs or their presence in tumours is not affected by Usp18. However, a higher percentage of activated T cells was observed in PyVmT/Usp18 KO tumours (Sup Information Fig. 1A).

Referee #3:

The manuscript by Burkart et. al., shows that ablation of USP18 in mammary epithelial cells generates an antitumorigenic microenvironment resulting in reduced tumor burden in the PyVMT mammary carcinoma model. The observations made in the paper are intriguing and are suggestive of activation of a Th1 response in USP18 KO tumors. The manuscript is well written and is able to convey the significance of the study. However, there are several aspects in the paper, which require further clarification in order to validate and strengthen the conclusions made in this manuscript.

Minor comments:

Figure 2G should include PyVMT WT MECs also in the invasion assay.

We are currently performing more studies to address the role of Usp18 in tumour invasion and the work will be reported in the future. Supporting Information Fig 2C shows that WT MECs behave no different from rescued Usp18 KO + Usp18 MECs *in vivo* suggesting that WT cells may behave similar in *in vitro* invasion assays too.

Fig 2E requires quantitation and power calculations.

Power calculations were added to Fig 2E and all other pictures that show microcopy photographs.

Fig. 3E should be instead included in fig.1

Since there is no panel E in figure 3 or supporting figure 3, could we receive additional information?

Fig. 4B should indicate the duration of MEC culture to generate supernatant.

Requested information was added to Material and Methods section.

Fig. 6C should be quantitated for phosphorylated STAT1 with reference to tubulin as well as STAT1. The authors should discuss the source of IFN-λ in the tumor microenvironment as they claim it to be the most sensitive pathway for USP18 ablation. If this is indeed true, then cellular identification of the source is required.

Quantification was added to the figure. We also added a section to the discussion about potential sources of IFN-λ production as suggested by the reviewer.

Fig 6B CXCL10 levels are inconsistent with that shown in Fig 4A.

It would be difficult to compare Cxcl10 transcript levels between tumour and cell line derived cDNAs since tumour lysates contain proteins and cytokines from a variety of cells that may affect Cxcl10 expression. We believe that the important finding is the similar trend in both systems.

Major comments:

Fig. 1 does not show if there is overall survival differences in USP18 KO mice as compared to WT PyVMT. This is required.

We added a figure showing survival differences of PyVmT/Usp18 KO and PyVmT/WT mice (Fig 1B). Since tumour growth in Usp18 KO mice is drastically reduced we chose an average tumour diameter of 0.5 cm as artificial endpoint to generate a survival curve. In addition, within the limited time available for revision experiments we were only able to generate three PyVmT/Usp18 KO mice for these studies. As expected PyVmT/WT mice reached study endpoint much earlier than PyVmT/Usp18 KO mice.

Fig. 2 touches upon the mechanism of reduced tumor burden in USP18 KO mice. However, the assays are inappropriate. Histochemical evaluation of primary tumor sections is required to evaluate changes in proliferation and/or cell death. Invasion assays and metastasis rate is neither informative nor helpful in this context. Differences in vessel density would be better supported by analyzing the expression levels of angiogenesis regulators in tumors.

As suggested, Fig 2 has been modified accordingly. TUNEL and Ki67 staining of WT and Usp18 KO tumour sections were added. PCNA and Caspase-3 Western Blotting figure was removed.

Fig.3 should include additional cell surface markers to strengthen data shown, e.g., CD11b, Ly6C, and Ly6G add resolution to the myeloid cell repertoire, which is not represented comprehensively by F4/80. Authors concluded CD4 T cells as the effector cell population based on their increased representation in cellular composition of USP18 KO tumors. CD4 T cells may play a critical role in USP18 KO tumors but this requires additional analyses to support, including depletion of all CD4+ T cells as opposed to mere depletion of T reg cells. In addition, IF studies to examine the FoxP3 and CD25 status of CD4 T cells would be helpful. Mere quantitation of CD8 T cells is not be conclusive regarding their influence on tumor growth. These must be evaluated IFN-γ production, granzyme B, CD25 and proliferation.

We previously analysed tumour lysates for $CD11b^{+}/Gr\text{-}1^{+}$ cells (myeloid derived suppressor cells) but dit not include the data in the original manuscript. We now added these data to Fig. 3B. Furthermore, to support the role of $CD4^{+}$ T cells in Usp18 dependent tumour development, as suggested, CD4+ T cell

depletion assay was conducted and results are shown as Fig 3D. We also added a panel to the Supplement showing activation status of $CD4^+$ and $CD8^+$ T cells (Supporting Information Fig. 1A). However, since our manuscript revolves around $CD4^+$ T cells as the main effector cells we did not characterize $CD8^+$ T cells in Usp18 KO mice further but agree with the reviewer that a more detailed evaluation of this population is desirable. Current efforts in our laboratory focus on the effect of Usp18 on immunophenotypes and cytotoxicity of $CD8^+$ T cells as well as polarization of Macrophages.

Fig. 5 shows that CXCL10 is a MEC-derived paracrine regulator of the tumor microenvironment. This data would be improved by analysis of vessel density, proliferation and apoptosis status in tumors derived from USP18 KO MECs with CXCl10 knockdown. Moreover, tumor cell composition should also be analyzed in these tumors with a focus on CD4 T cells. It is obvious to inquire here if recombinant CXCl10 administration in WT-PYMT mice or ectopic expression of CXCL10 in MECs will cause stunted tumor growth. Moreover, since CXCL10 is an angiostatic chemokine, ex vivo endothelial invasion assays should be considered.

This is a good point. As kindly suggested by the reviewer, we performed the requested IHC staining on control and Cxcl10 knockdown tumours and analysed CD4+ T cell population by flow cytometry (Supporting Information Fig 3). Since similar antitumour effects of Cxcl10 have been reported (Wang P et al. Cancer Immunol Immunother, 59, 1715-1726 and Tominaga M et al. Cancer Gene Ther, 14, 696-705), we did not investigate the effect of ectopic expression or administration of Cxcl10 on mammary tumour growth but mentioned these reports in the discussion.

Fig 6 shows inhibition of STAT1 activation by USP18 expression and increased CXCL10 production when IFN is applied to USP18 KO MECs. There is no data provided to indicate that these changes are also observed in vivo in the tumors. Tumor sections should be quatitatively evaluated for CXCL10 and pSTAT1 presence and co-localization. Lack of this data necessitates removal of Fig 6 and mere mention of it as a possible scenario in the conclusion.

We appreciate these comments. Since we could not find compatible antibodies of p-Stat1 and Cxcl10 to perform co-immunostaining in the same fixation with numerous attempts, we added Western Blotting data for pStat1 and Cxcl10 on tumour lysates from PyVmT/Usp18 WT and PyVmT/Usp18 KO mice (Fig. 6D). Results show a correlation between high p-STAT1 and Cxcl10 in Usp18 KO tumours.

In conclusion, we are thankful to all three reviewers for their enthusiasm for our manuscript and for raising a series of constructive criticisms to further improve our work. We have clarified a number of issues raised by the reviewers and performed additional experiments to support our conclusions. We thus hope that the reviewers now find our manuscript suitable for publication at *EMBO Molecular Medicine*.

2nd Editorial Decision 26 March 2013

Thank you for the submission of your revised manuscript to EMBO Molecular Medicine. We have now received the enclosed reports from the referees that were asked to re-assess it. As you will see the reviewers are now globally supportive and I am pleased to inform you that we will be able to accept your manuscript pending the following final amendments:

-In light of Referee #2's comments, please discuss the biological significance of pro- or anti-tumoral effects of CD4 T cells according to Usp18 status.
-Please carefully proof-read and spell-check your article for clarity.
-However, regarding the other requirements of this referee, we leave it up to you to comply or leave the data as it is.

Please submit your revised manuscript within two weeks.

I look forward to reading a new revised version of your manuscript as soon as possible.

***** Reviewer's comments *****

Referee #1 (Comments on Novelty/Model System):

This work is paramount for our understanding of unique cytokine contribution to the mammary tumorigenesis throgh the tumor microenivronment-based mechanistic regulation

Referee #1 (General Remarks):

Authors have carefully revised this work. This strengthened manuscript will be of great interest to the Journal's readership.

Referee #2:

The authors have addressed adequately the several concerns and suggestions of the referees. A few questions remain unanswered. For example, it is not clear whether increased CXCL10 production by USP18-deficient cancer cells is directly responsible for the enhanced recruitment of T-cells to the tumors. Indeed, as previously mentioned, this may also represent a tumor-stage specific effect. Nevertheless, the new data in Fig 3D satisfactorily illustrate the involvement of CD4+ T-cells in the observed phenotype. The data may be combined into a single graph, and the white bars (anti-CD4) compared side by side to show that tumor growth after CD4+ T-cell depletion is similar in "rescued" (i.e., USP18-KO + USP18) and USP18-KO cancer cell-bearing mice. The findings in Fig 3D are indeed very interesting, as they also show that CD4+ T-cells are either pro- or antitumoral depending on the USP18 status of the cancer cells. While additional studies would certainly be beyond the remit of this article, I believe that the authors should further speculate on the biological significance of these findings.

I note that the CD31 data (blood vessel density) in both Figures 2D and S3B lack quantification. If the authors want to stress the antiangiogenic effect of USP18 deficiency, some quantification of the data should be provided.

Data in Fig. 1C are not essential and should be removed.

The English is at times poor and the manuscript would benefit from a thorough revision.

2nd revision - authors' response 04 April 2013

Thank you very much for accepting our revised manuscript, "Usp18 deficient mammary epithelial cells create an antitumour environment driven by hypersensitivity to IFN-λ and elevated secretion of Cxcl10". We are pleased to see that only minor changes are required for publication.

As you suggested, we carefully proof-read and spell-checked the manuscript. We also included more text to discuss the role of Usp18 as a regulator of pro- or antitumoral effects of CD4+ T cells. These additional sections are in blue font in the text.

Furthermore, we added a new figure to the manuscript (Fig. 7). This figure summarizes our findings and underlines the importance of both IFN-λ signaling and presence of CD4+ T cells for tumor inhibition by Usp18 deficient MECs. Figures 1 - 6 were not changed.

© EMBO
