# Supplementary material for: Usp18 deficient mammary epithelial cells create an antitumour environment driven by hypersensitivity to IFN-λ and elevated secretion of Cxcl10
Source: EMBO Mol Med. 2013 May 16;5(7):967–82. doi: 10.1002/emmm.201201864 (PMC3721472; doi:10.1002/emmm.201201864)
Supplement: Supplementary file 2 [file emmm0005-0967-SD2.pdf]

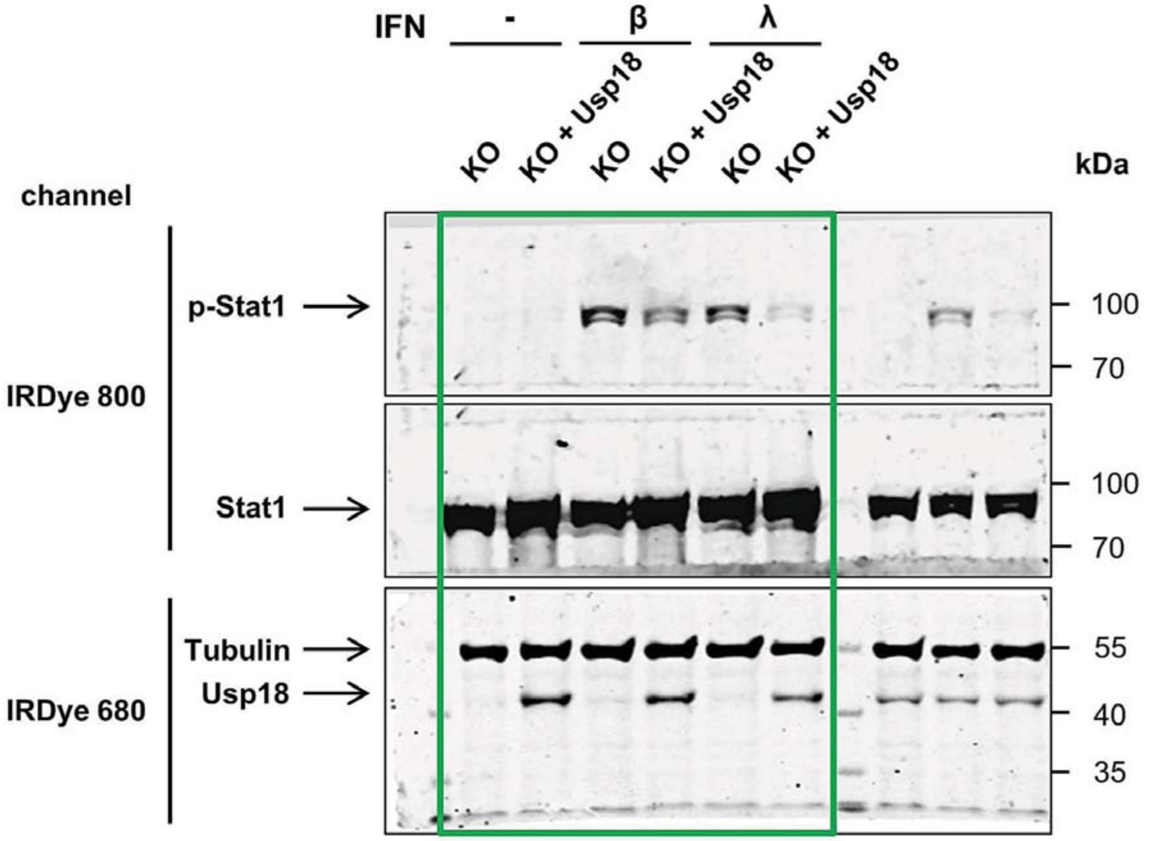

Li-Cor Odyssey scans of Figure 6C

After transfer nitrocellulose membranes were cut into small strips to save antibody solution and allow simultaneous probing for multiple proteins. Shown are unprocessed scans of each strip. The green box marks the lanes shown in report. Channels for detection of fluorophore-conjugated secondary antibodies are shown on the left. Only the channel used for detection of IRDye 680 can sufficiently detect Coomassie Blue based protein markers.

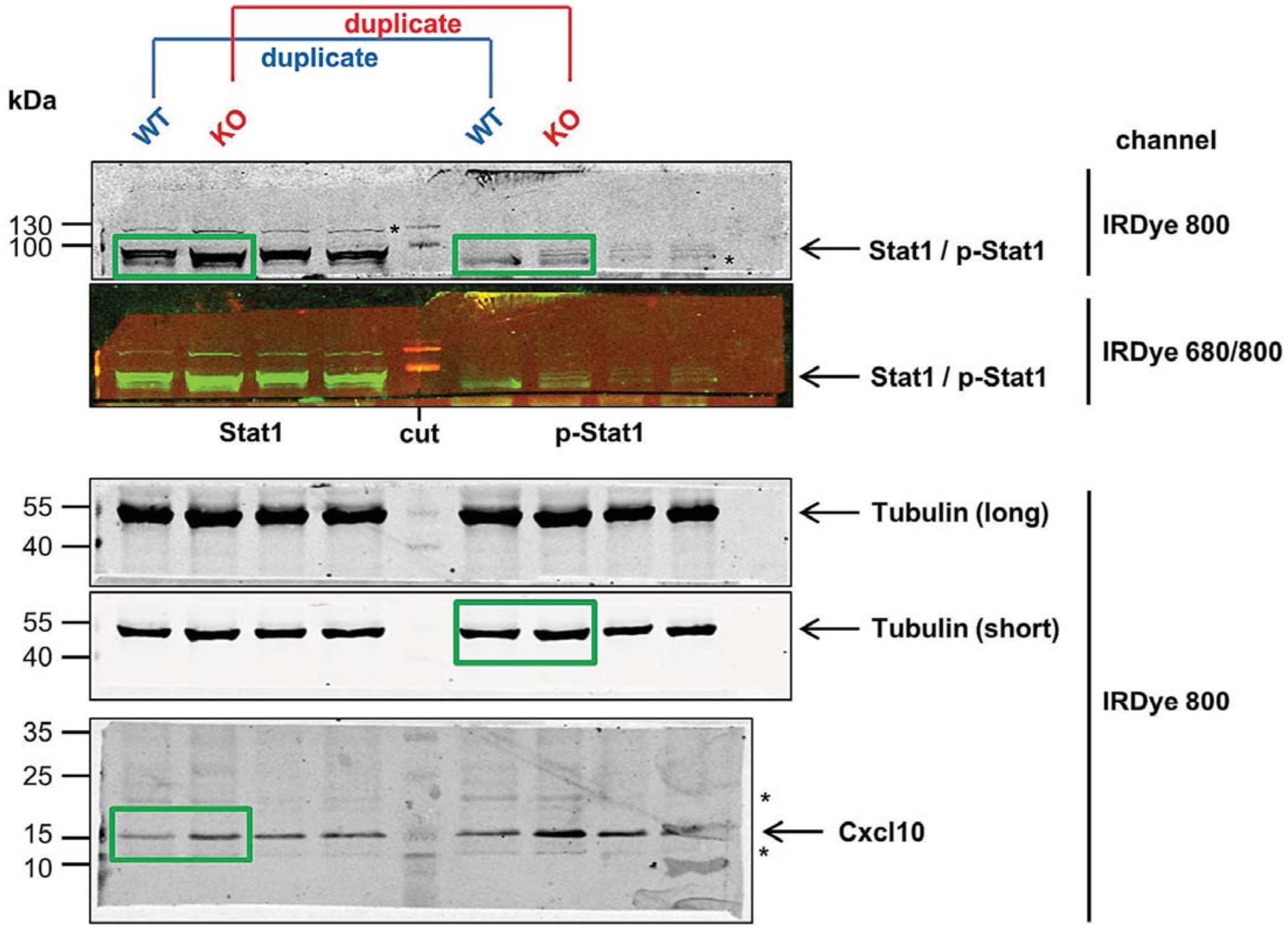

Li-Cor Odyssey scans of Figure 6D

After transfer nitrocellulose membranes were cut into small strips to save antibody solution and allow simultaneous probing for multiple proteins. Shown are unprocessed scans of each strip. WT and KO sample was loaded in duplicate to allow simultaneous probing for Stat1 and p-Stat1. Two colour image of membranes probed for Stat1 and p-Stat1 are shown to better visualize the area where membrane was cut. Equal loading of duplicates confirmed by Tubulin. The areas highlighted green are shown in report. Channels for detection of fluorophore-conjugated secondary antibodies are shown on the right. Unspecific bands are indicated by asterisks.

Figure 6D, Burkart et al

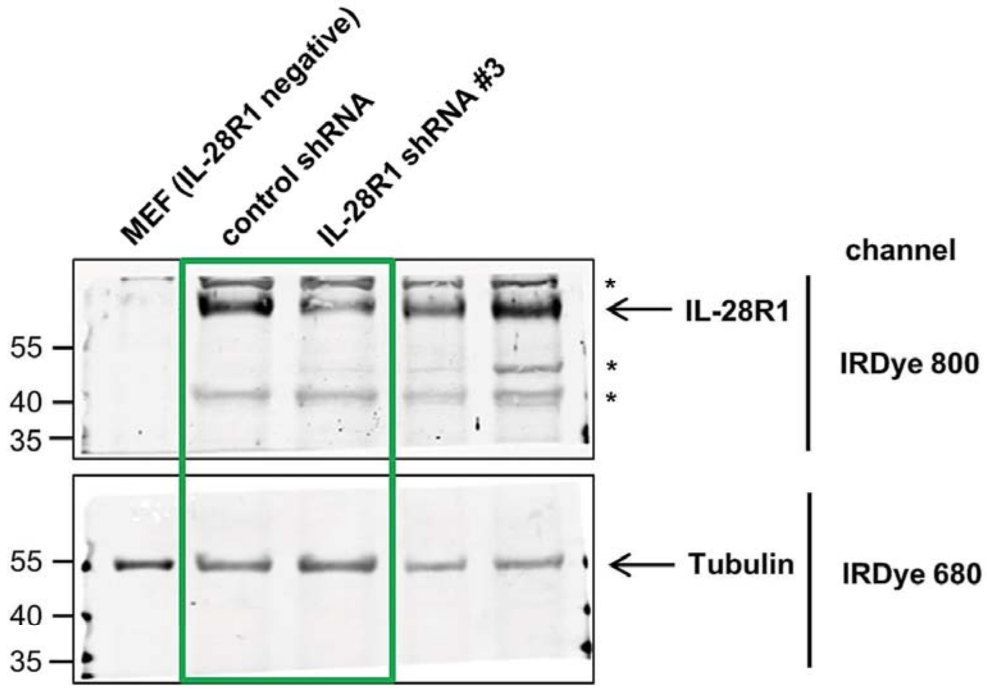

***Li-Cor* Odyssey scans of Figure 6E**

Protein lysate form murine embryonic fibroblasts (MEF) was used as negative control for IL-28R1 detection but only lanes marked with green box are shown in report. After transfer nitrocellulose membranes were cut into small strips to save antibody solution and allow simultaneous probing for multiple proteins. Shown are unprocessed scans of each strip. Channels for detection of fluorophore-conjugated secondary antibodies are shown on the right. Only the channel used for detection of IRDye 680 can sufficiently detect Coomassie Blue based protein markers. Unspecific bands are indicated by asterisks.
