# Supplementary material for: Usp18 deficient mammary epithelial cells create an antitumour environment driven by hypersensitivity to IFN-λ and elevated secretion of Cxcl10
Source: EMBO Mol Med. 2013 May 16;5(7):967–82. doi: 10.1002/emmm.201201864 (PMC3721472; doi:10.1002/emmm.201201864)
Supplement: Supplementary file 3 [file emmm0005-0967-SD3.pdf]

Supporting Information

Usp18 deficient mammary epithelial cells create an antitumour environment driven by hypersensitivity to IFN-λ and elevated secretion of Cxcl10 (Burkart *et al*)

Table of Contents:

Supporting Information Fig 1

Supporting Information Fig 2

Supporting Information Fig 3

Supporting Information Fig 4

Supporting Information Fig 5

Supporting Information Fig 6

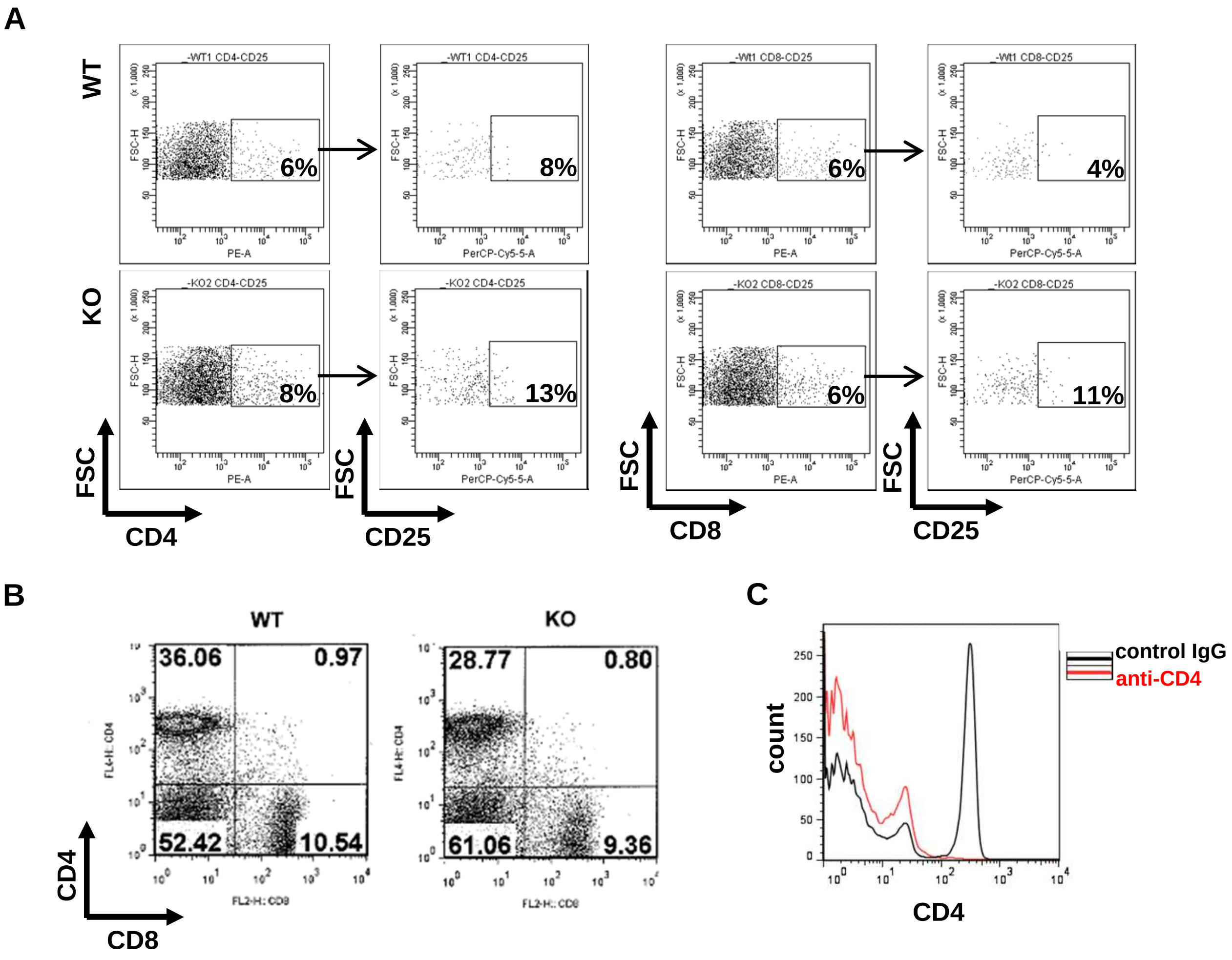

Supporting Information Fig 1: Involvement of CD4$^+$ cells in PyVmT driven tumourigenesis.

A) Tumor single cell suspensions from PyVmT/Usp18 WT and PyVmT/Usp18 KO mice were analysed by flow cytometry for CD4$^+$/CD25$^+$ and CD8$^+$/CD25$^+$ double positive cells. B) Spleens from Usp18 WT or KO mice of 8 weeks of age were harvested and single cell suspensions analysed for CD4$^+$ and CD8$^+$ cells by flow cytometry. C) FACS data showing efficient depletion of CD4$^+$ cells in FVB WT mice two days after intraperitoneal injection of 100µg anti-CD4 antibody.

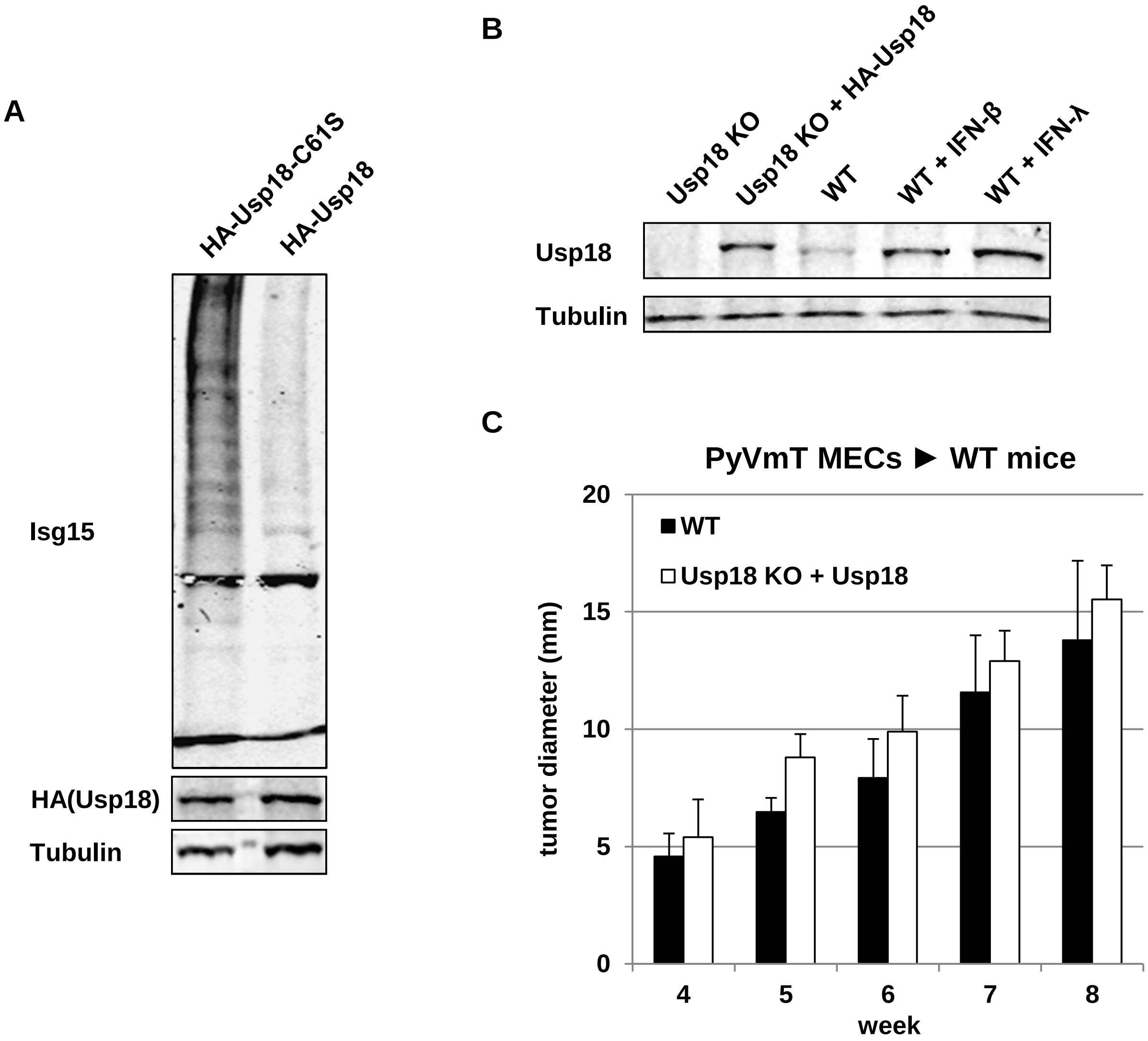

Supporting Information Fig 2: Validation of expression levels and activity of retrovirally expressed Usp18 variants.

A) Expression levels and enzyme activity of HA-Usp18-C61S and HA-Usp18 introduced into PyVmT/Usp18 KO MECs analyzed by anti-HA and anti-Isg15 Western Blotting. Tubulin was used as loading control. B) Expression of retrovirally expressd HA-Usp18 compared to endogenous levels in WT MECs with or without IFN treatment (24h). Anti-mUsp18 antibody was used for detection of Usp18 protein. Tubulin was used as loading control. C) Mammary tumor growth in WT mice injected with either WT MECs or Usp18 KO + Usp18 MECs. Tumor growth was monitored by weekly measurings of tumor diameter with a caliper.

A

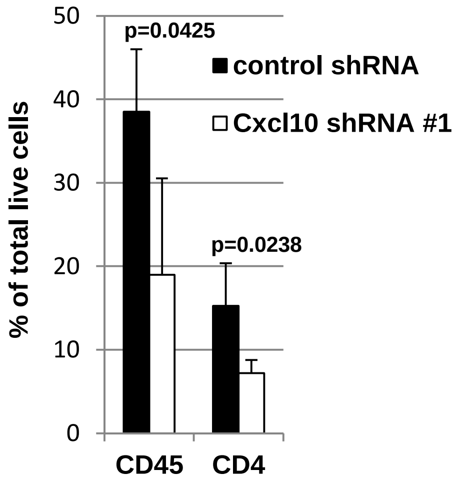

B

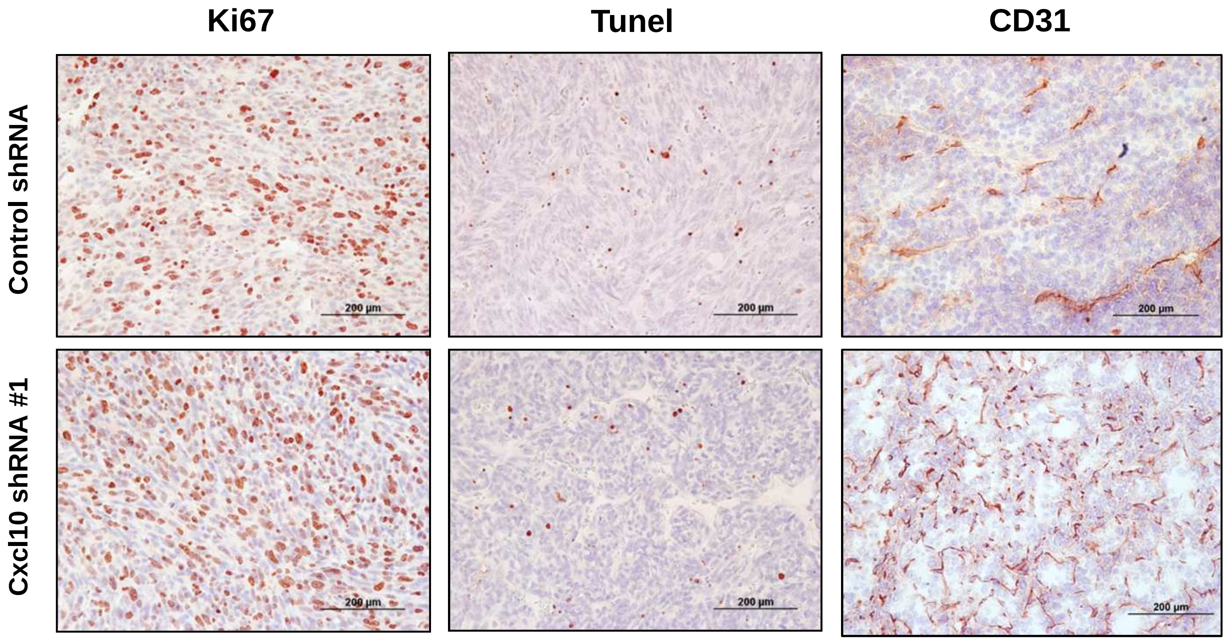

Supporting Information Fig 3: Analysis of tumors derived from Cxcl10 knockdown PyVmT/Usp18 KO MECs.

A) Flow cytometric analysis of single cell suspensions from tumors derived from PyVmT/Usp18 KO Cxcl10 shRNA #1 MECs. Total percentage of leukocytes and $CD4^{+}$ T cells present in the tumor was determined. Control shRNA , n=3; Cxcl10 shRNA, #1 n=3). B) Immunohistochemical analysis of paraffin-embedded tumour tissues from control and Cxcl10 shRNA #1 PyVmT/Usp18 KO tumors. Images are 200x with 200 µm scale bar.

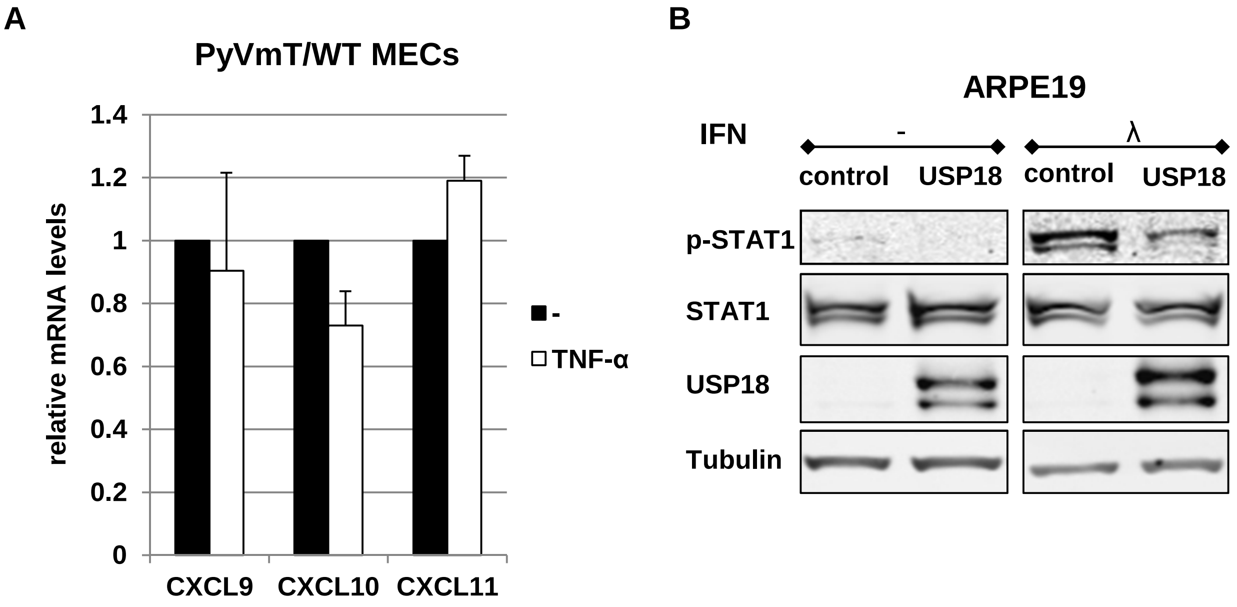

Supporting Information Fig 4: TNF-α does not induce Cxcr3 ligands in PyVmT MECs and Usp18 is a negative regulator of IFN-λ signaling in human epithelial cells.
A) Induction of Cxcr3 ligands upon TNF-α treatment in transduced PyVmT MECs was analysed by qRT-PCR. Relative means from three independent experiments normalized to untreated cells are shown. B) Human retinal pigment epithelial ARPE19 cells stably transduced with control or USP18 expressing retroviruses were left untreated or treated with IFN-λ for 15 min. Cells were harvested, lysed and analysed for USP18, STAT1 and phosphorylated STAT1 levels by Western blotting. Tubulin was used as loading control.

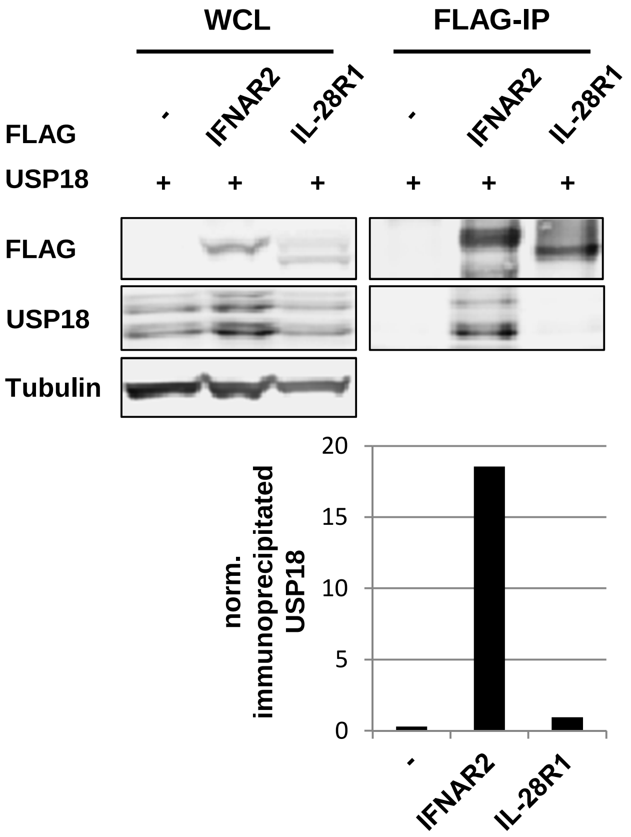

Supporting Information Fig 5: Usp18 does not associate with IL-28R1 in co-immunoprecipitation experiments.
Protein lysates from transfected 293T cells were subjected to FLAG-IP and immunoprecipitated USP18 detected by Western blotting. Amount of immunoprecipitated USP18 normalized to tubulin and expression levels of USP18 (input) was quantified with the LICOR Odyssey system.

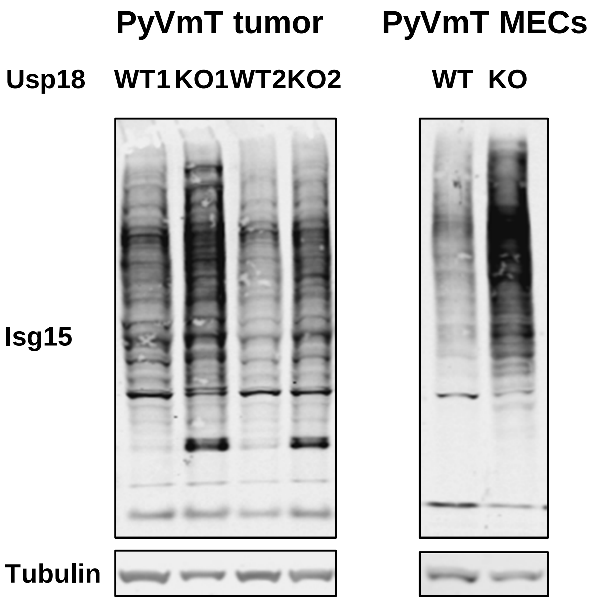

Supporting Information Fig 6: Mammary tumours and isolated PyVmT MECs show constitutively high levels of ISGylated protein, which are further elevated in the absence of Usp18.
Protein lysates from PyVmT tumours and PyVmT MEC cell lines were analysed for ISGylation levels by anti-Isg15 Western blotting. Tubulin was used as loading control.
